# Structural Engineering of Low‐Dimensional Metal–Organic Frameworks: Synthesis, Properties, and Applications

**DOI:** 10.1002/advs.201802373

**Published:** 2019-04-18

**Authors:** Wenxian Liu, Ruilian Yin, Xilian Xu, Lin Zhang, Wenhui Shi, Xiehong Cao

**Affiliations:** ^1^ College of Materials Science and Engineering Zhejiang University of Technology 18 Chaowang Road Hangzhou Zhejiang 310014 P. R. China; ^2^ Center for Membrane Separation and Water Science & Technology Ocean College Zhejiang University of Technology 18 Chaowang Road Hangzhou Zhejiang 310014 P. R. China; ^3^ Huzhou Institute of Collaborative Innovation Center for Membrane Separation and Water Treatment Zhejiang University of Technology Huzhou Zhejiang 313000 P. R. China; ^4^ State Key Laboratory Breeding Base of Green Chemistry Synthesis Technology Zhejiang University of Technology 18 Chaowang Road Hangzhou Zhejiang 310032 P. R. China

**Keywords:** catalysts, electrode materials, low‐dimensional nanomaterials, metal–organic frameworks, sensors

## Abstract

Low‐dimensional metal–organic frameworks (LD MOFs) have attracted increasing attention in recent years, which successfully combine the unique properties of MOFs, e.g., large surface area, tailorable structure, and uniform cavity, with the distinctive physical and chemical properties of LD nanomaterials, e.g., high aspect ratio, abundant accessible active sites, and flexibility. Significant progress has been made in the morphological and structural regulation of LD MOFs in recent years. It is still of great significance to further explore the synthetic principles and dimensional‐dependent properties of LD MOFs. In this review, recent progress in the synthesis of LD MOF‐based materials and their applications are summarized, with an emphasis on the distinctive advantages of LD MOFs over their bulk counterparties. First, the unique physical and chemical properties of LD MOF‐based materials are briefly introduced. Synthetic strategies of various LD MOFs, including 1D MOFs, 2D MOFs, and LD MOF‐based composites, as well as their derivatives, are then summarized. Furthermore, the potential applications of LD MOF‐based materials in catalysis, energy storage, gas adsorption and separation, and sensing are introduced. Finally, challenges and opportunities of this fascinating research field are proposed.

## 1 Introduction

As a typical class of crystalline materials, metal‐organic frameworks (MOFs) have emerged as one of the most attractive research fields in recent years.^1^, ^2^, ^3^, ^4^, ^5^, ^6^, ^7^, ^8^ MOFs which are assembled by metal ions/clusters and organic ligands possess diversified composition, tailorable structure, large surface area, and uniform cavity. Such unique structural and compositional features make MOF‐based materials promising for a wide range of applications, such as gas storage and separation, catalysis, sensors, and drug delivery.^9^, ^10^, ^11^, ^12^, ^13^, ^14^, ^15^, ^16^, ^17^, ^18^, ^19^, ^20^ Great efforts have been also devoted to preparing nanoscale MOF crystals with controllable size and morphology, in order to achieve enhanced performance in certain applications.^21^, ^22^, ^23^, ^24^ Similar to the size‐dependent properties which are well‐known for nanomaterials, reducing size of MOF crystals may bring enlarged specific surface area and increased density of active sites for MOFs, leading to improved performance.^25^, ^26^, ^27^, ^28^, ^29^

Low‐dimensional (LD) nanomaterials are of particular interest due to their anisotropic‐tunable properties, which greatly influence their performances.^30^, ^31^, ^32^, ^33^, ^34^, ^35^, ^36^, ^37^ Since the discovery of carbon nanotubes (CNTs) in the early 1990s, 1D nanostructures, such as nanowires, nanotubes, and nanorods, have been extensively investigated because of their unique geometrical and electronic characteristics.^38^, ^39^, ^40^ As another typical LD materials, 2D nanomaterials, such as 2D nanosheets of layered double hydroxides (LDHs), transition metal dichalcogenides (TMDs), hexagonal boron nitride, and MXenes, have attracted widespread attention since the discovery of graphene.^41^, ^42^, ^43^, ^44^, ^45^, ^46^, ^47^

**Figure 1:**
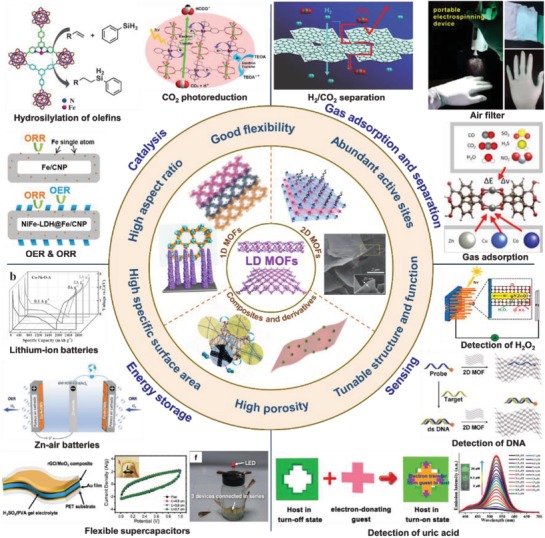
Unique properties of low‐dimensional (LD) MOF‐based materials and their applications. Top left: some representative examples of LD MOF‐based materials used as catalysts. Hydrosilylation of olefins. Reproduced with permission.^64^ Copyright 2016, Wiley‐VCH. OER & ORR. Reproduced with permission.^65^ Copyright 2018, Wiley‐VCH. CO_2_ photoreduction. Reproduced with permission.^66^ Copyright 2015, Royal Society of Chemistry. Top right: some representative examples of LD MOF‐based materials used in the field of gas adsorption and separation. H_2_/CO_2_ separation. Reproduced with permission.^67^ Copyright 2017, Wiley‐VCH. Air filter. Reproduced with permission.^70^ Copyright 2016, American Chemical Society. Gas adsorption. Reproduced with permission.^68^ Copyright 2017, American Chemical Society. Bottom left: some representative examples of LD MOF‐based materials used in the field of energy storage. Flexible supercapacitors. Reproduced with permission.^73^ Copyright 2015, Wiley‐VCH. Zn‐air batteries. Reproduced with permission.^71^ Copyright 2018, Wiley‐VCH. Lithium‐ion batteries. Reproduced with permission.^72^ Copyright 2016, Wiley‐VCH. Bottom right: some representative examples of LD MOF‐based materials used as sensor. Detection of H_2_O_2_. Reproduced with permission.^74^ Copyright 2013, American Chemical Society. Detection of DNA. Reproduced with permission.^75^ Copyright 2015, Wiley‐VCH. Detection of uric acid. Reproduced with permission.^69^ Copyright 2017, Royal Society of Chemistry. Middle: some representative examples of LD MOF‐based materials. Bottom left image in 1D MOFs panel. Reproduced with permission.^91^ Copyright 2017, Elsevier. Top right image in 1D MOFs panel. Reproduced with permission.^97^ Copyright 2009, Wiley‐VCH. Top left image in 2D MOFs panel. Reproduced with permission.^168^ Copyright 2016, Wiley‐VCH. Bottom right image in 2D MOFs panel. Reproduced with permission.^82^ Copyright 2014, Springer Nature. Left image in composites and derivatives panel. Reproduced with permission.^112^ Copyright 2014, American Chemical Society. Right image in composites and derivatives panel. Reproduced with permission.^214^ Copyright 2016, Wiley‐VCH.

**Figure 2:**
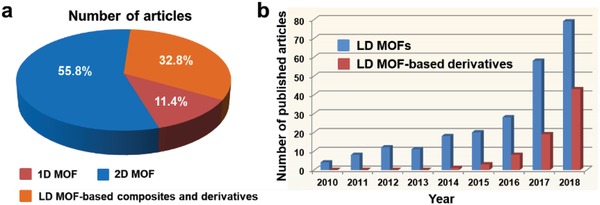
The summary of publications regarding to LD MOF‐based materials in recent years (2010–2018), as searched by “Web of Science” (until 2018 December 20).

More recently, LD MOFs have attracted intensive interest and been investigated in various applications.^48^, ^49^, ^50^ These types of MOFs, such as 1D single‐walled MOF nanotubes, 1D hexagonal MOF nanorods, ultrathin 2D MOF nanosheets,^51^, ^52^, ^53^ have similar morphological features to those of the other 1D or 2D nanomaterials, which endows them with distinctive dimensional‐dependent properties including high aspect ratio and abundant accessible active sites. Besides, the intrinsic characteristics of MOFs allow the facile tuning of their microstructures and properties for LD MOFs. To achieve effective and controllable preparation of LD MOFs, a large number of synthetic methods have been developed, such as template method, ultrasonic exfoliation, surfactant‐assisted growth, three‐layer method, etc.^54^, ^55^, ^56^ Additionally, various LD MOF‐based composites and their derived hybrid nanomaterials with multifunctions have also been successfully prepared in recent years.^57^, ^58^, ^59^ Owing to their unique structures, LD MOFs are promising for a variety of applications, including gas adsorption and separation, heterogeneous catalysis, energy storage and conversion, and so on (**Figure** **1**).^60^, ^61^, ^62^, ^63^, ^64^, ^65^, ^66^, ^67^, ^68^, ^69^, ^70^, ^71^, ^72^, ^73^, ^74^, ^75^ Although the advantages and challenges of 1D metal‐organic nanotubes^76^ and 2D MOF nanosheets^48^, ^50^, ^56^ have been well summarized by several previous review articles, the research field of LD MOFs is currently at a stage of rapid development, as indicated by annually increased amount of publications (**Figure** **2**). Therefore, a latest review article is highly demanded, which helps the readers to have a comprehensive understanding of the recent progress of LD MOFs regarding to their rational design strategies, controllable synthetic methods and potential applications. More importantly, this review helps readers to gain a better insight into the advantages of LD MOFs over their bulk counterparts.

In this review, an overview of the progress on synthesis and application of LD MOF materials is presented, including 1D MOFs, 2D MOFs, and LD MOF‐based composites, as well as their derivatives. This review begins with the introduction and discussion of the unique advantages of LD MOF‐based materials. Then, we highlight the typical synthetic methods for LD MOF‐based materials, as well as their derivatives, followed by a summary of their applications in catalysis, energy storage, gas adsorption and separation, and sensing. Finally, the challenges and perspectives of this fascinating research field are discussed.

## 2 Distinctive Advantages of LD MOFs

In general, the dimensions of MOFs have a profound influence on their physicochemical properties.^30^, ^77^ Compared to their bulk counterparts, LD MOFs possess unique structural advantages, such as good flexibility and abundant surface active sites, which endow them with new functions. The critical strategy of the formation of LD MOFs is to confine the growth of MOF crystals in one or two directions. **Table** **1** summarizes the dimensional‐dependent properties of LD MOF‐based materials along with their corresponding anisotropic growth process. Obviously, the dimensional engineering of MOF crystals provides LD MOFs with enhanced properties, which leads to improved performances in certain applications. In this section, we use comparative examples to demonstrate the superiority of LD MOFs compared to their bulk counterparts.

**Table 1:** Comparison of performances of MOF‐based materials with various dimensions and their corresponding synthetic methods

Nanomaterials	Feature	Methods	Application and property	Ref.
			OER	151
NiCo MOF	2D	Ultrasonic method	*E*_onset_: 1.42 V (vs RHE)	
	Bulk	Hydrothermal method	*E*_onset_: 1.48 V (vs RHE)	
			OER	278
NiFe‐MOF	2D	In situ growth on nickel foam)	η_10_: 240 mV (vs RHE)	
	Bulk	Solvothermal method	η_10_: 318 mV (vs RHE)	
			ORR	112
ZIF‐8 derived porous carbon	1D	Template synthesis followed by annealing	*E*_half‐wave_: −0.161 V (vs Ag/AgCl)	
	Bulk	Template‐free synthesis followed by annealing	*E*_half‐wave_: −0.367 V (vs Ag/AgCl)	
			Supercapacitors	89
Cu–CAT MOF	1D	With carbon fiber paper	*C*_s_ (0.5 A g^−1^): 202 F g^−1^	
	Bulk	Without carbon fiber paper	*C*_s_ (0.5 A g^−1^): 80 F g^−1^	
			Photocatalysis	79
Zr MOF	2D	Pseudo‐assembly/disassembly strategy	Absorbance at 419 nm: 0.17	
	Bulk	Solvothermal method	Absorbance at 419 nm: 0.08	
			C—H bond catalytic oxidation	125
Cu MOF	1D	Crystal phase transformation using 1‐methylimidazole	Conversion: 94.3% Selectivity:96.1%	
	Bulk	Crystal phase transformation using BTC	Conversion: 87.0% Selectivity:93.9%	
			CO_2_ photoreduction	66
Ru‐MOF	0D	Solvothermal	Product formation rate: 52.7 µmol g^−1^ h^−1^	
	Nano flowers	Kinetic control	Product formation rate: 77.2 µmol g^−1^ h^−1^	
	Bulk	Solvothermal	Product formation rate: 30.6 µmol g^−1^ h^−1^	
			Catalytic reduction of 4‐nitrophenol	226
MOF‐2	0D	Solvothermal method	Rate constant: 0.21 min^−1^	
	2D	Kinetic control solvothermal method	Rate constant: 0.41 min^−1^	
			DNA detection	75
Cu‐TCPP	2D	Surfactant‐assisted method	Quenching efficiencies for P1:89%	
	Bulk	Solvothermal method	Quenching efficiencies for P1:19%	
			Gas adsorption	176
Zr‐BTB MOF	2D	Continuous microdroplet flow method	CO_2_: 12 mmol g^−1^; CH_4_: 15 mmol g^−1^	
	Bulk	Solvothermal method	CO_2_: 4 mmol g^−1^; CH_4_: 6 mmol g^−1^	
			Lithium–sulfur batteries	245
ZIF‐8 derived porous carbon	2D	Organic‐solvent‐free approach followed by annealing	785 mA h g^−1^ at 2 C	
	Bulk	Organic‐solvent approach followed by annealing	399 mA h g^−1^ at 2 C	
			Supercapacitor	169
PPF‐3 derived CoS_1.097_/N‐C	2D	Surfactant‐assisted approach followed by annealing	360.1 F g^−1^ at 1.5 A g^−1^	
	Bulk	Solvothermal method followed by annealing	10.6 F g^−1^ at 1.5 A g^−1^	
			ORR	152
ZIF‐67 derived Co/N‐C	2D	Salt‐template method followed by annealing	*E*_half‐wave_: 0.869 V (vs RHE)	
	Bulk	Solvothermal method followed by annealing	*E*_half‐wave_: 0.832 V (vs RHE)	
			Lithium ion battery	305
Ge MOF derived GeO_2_ nanosheets	2D	Solvothermal method	1393 mAh g^−1^ after 350 cycles	
	Bulk	Commercial production	403 mAh g^−1^ after 350 cycles	

One of the most attractive properties of LD MOFs compared to their bulk counterparts is their highly exposed surface area. On the one hand, the enlarged accessible surface area for LD MOFs can shorten the diffusion distances of reactants and/or products. On the other hand, LD MOFs possess improved atomic utilization of active sites, leading to much enhanced performances in applications like catalysis and sensing.^78^, ^79^, ^80^ As demonstrated by Wang and co‐workers, the ultrathin 2D Zr‐TCPP(Ni) nanosheets showed much higher catalytic activity than their bulk counterparts in the photocatalytic oxidation of 1,5‐dihydroxynaphthalene to juglone.^79^ Furthermore, the improved fluorescence quenching efficiency of Texas red‐labeled single‐stranded DNA (89%) was demonstrated by using Cu‐TCPP nanosheets, while the efficiency of bulk Cu‐TCPP MOFs was only 19%.^75^ The enhancement in fluorescence quenching performance for Cu‐TCPP nanosheets may be attributed to the 2D structures, which possess higher density of accessible surface sites as compared to their bulk counterparts, thus facilitating energy and electron transfer between fluorescent molecules and quenching agent.

Benefiting from the strong coordination bonds and reduced size in one or two dimensions, LD MOFs, especially ultrathin 2D MOF nanosheets and 1D MOF nanofibers, usually exhibit good mechanical property and high flexibility. These properties facilitate the fabrication of functional MOF thin films for the applications of gas storage and separation. For example, Zamora and co‐workers characterized the mechanical properties of 2D laminar MOF nanosheets, i.e., [Cu(m‐pym_2_S_2_)(µ‐Cl)]*_n_*, by using nanoindentation technique.^81^ The Young's modulus of the MOF nanosheets is calculated to be 5 GPa. Although this value is much lower than that of graphene oxide nanosheets (≈200 GPa), it is high enough to form a robust and freestanding MOF thin film with good photoluminescence property. Besides, the good flexibility and mechanical properties of LD MOF films opened vast opportunities in the practical applications of air filter and optical devices.^67^, ^82^, ^83^ Moreover, the flexibility of LD MOFs also gives them the characteristics of morphological and structural transformation. For example, the morphology evolution of Mo‐polydopamine nanosheets to hierarchical flowers was demonstrated.^84^ Zhang and co‐workers also reported the preparation of 1D metal‐organic nanotube through rolling up a MOF layer.^85^

In addition, LD MOFs also possess promoted electrical conductivity due to shortened electron/charge transportation pathlength. It is well known that MOFs generally have low electrical conductivity owing to their chemical composition and crystalline features. However, recent works demonstrated the proper design of secondary building units (SBUs) and/ororganic ligands is able to prepare electrically conductive MOFs. Reducing the dimensions of the conductive MOFs further enhanced their electrical conductivity, making those MOFs promising for energy storage and sensing.^86^, ^87^, ^88^ As a typical example, Cu‐CTA nanowire array with conductivity of ≈20 S m^−1^ for an individual nanowire was grown on carbon paper, and then used as electrode for supercapacitor, which showed an areal capacitance of ≈22 µF cm^−2^.^89^ Compared to the electrode composed of Cu‐CTA powder, the electrode of MOF nanowire array exhibited good rate performance and cycling stability.

## 3 Synthetic Strategies for LD MOF‐Based Materials

### 3.1 Synthesis of 1D MOFs

**Figure 3:**
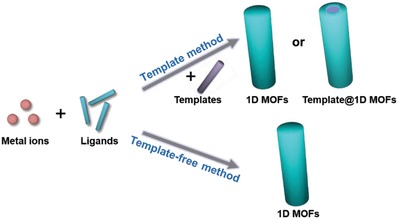
Schematic illustration of two typical preparation strategies for 1D MOFs.

To date, a variety of MOFs with 1D structure, such as nanorods,^53^, ^90^, ^91^, ^92^ nanotubes,^93^, ^94^, ^95^, ^96^, ^97^, ^98^, ^99^ and nanofibers,^52^, ^100^, ^101^, ^102^ have been synthesized and exhibited unique physicochemical properties.^38^, ^103^ As schematically illustrated in **Figure** **3**, the current preparation strategies for 1D MOF architectures can be generally classified into two types, i.e., template and template‐free methods. Generally, template method is able to effectively control the morphologies of MOFs, which have been widely utilized to prepare various 1D MOFs, such as hollow ZIF‐8 nanoworms,^104^ ZIF‐8 nanowires,^54^ and Cd‐BTC nanorods.^105^ On the other hand, template‐free method avoids the tedious process for the preparation of template and its subsequent removal step, which shows better universality and has received extensive attention from researchers. In this section, we summarize the recent progress of these two preparation methods of 1D MOFs, and briefly introduce the characteristics of these two methods through typical examples.

#### 3.1.1 Template Method

**Figure 4:**
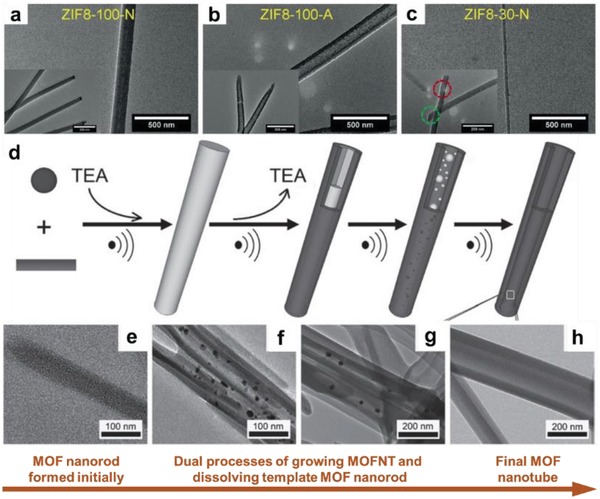
TEM images of a) ZIF8‐100‐N nanorods, b) ZIF8‐100‐A nanotubes, and c) ZIF8‐30‐N nanowires. Reproduced with permission.^54^ Copyright 2018, Wiley‐VCH. d) Schematic illustration of the preparation procedure for Cd‐BTC MOF nanotubes. e–h) TEM images of the obtained samples in different reaction stages. Reproduced with permission.^105^ Copyright 2011, Wiley‐VCH.

Template method is an effective technique to synthesize nanomaterials with controllable size and composition.^106^, ^107^, ^108^, ^109^, ^110^ Plenty of templates with rationally designed structures, e.g., polystyrene‐poly‐4‐vinylpyridine (PS‐b‐P4VP) block copolymer,^104^ cellulose,^111^ Te nanowires,^112^, ^113^ and ZnO nanorods,^74^, ^114^, ^115^, ^116^ can be used for the preparation of 1D MOFs. As one of the most widely used hard templates, track‐etched polycarbonate (PCTE) membrane have already been applied in the synthesis of inorganic nanowires.^117^, ^118^ Recently, single‐crystalline ZIF‐8 nanowires, polycrystalline ZIF‐8 nanorods and nanotubes have been successfully achieved by using commercial PCTE as the template.^54^ It was found that various experimental parameters, including pore size of template, concentration of reactant, and type of metal salt, have significant effects on the morphology of products. Typically, ZIF‐8 nanorods with length of about 6 µm were prepared by using Zn(NO_3_)_2_ and 2‐methylimidazole as reactants, and PCTE membrane with channel diameter of 100 nm as template (**Figure** **4**a). Interestingly, replacing the reactant with Zn(Ac)_2_, tubular ZIF‐8 was obtained (Figure 4b), which may be due to decreased growth rate of the ZIF‐8 crystal within the channels of PCTE. Moreover, single‐crystalline ZIF‐8 nanowires were prepared by using PCTE membrane with smaller channel size of 30 nm. The formation of nanowire structure could be attributed to the increased surface tension in the channel of PCTE, which leads to rapid dissolution and recrystallization of small ZIF‐8 seeds to form a large and stable crystal seed in a single channel (Figure 4c). Similarly, Peinemann and co‐workers reported the synthesis of 1D ZIF‐8 with worm‐like hollow structure relying on the template of a filament‐shaped amphiphilic block copolymer (BCP), i.e., PS‐*b*‐P4VP.^104^

Alternatively, synthesis of 1D MOFs has also been realized through self‐template approach. As a typical example, Zhu and co‐workers synthesized Cd‐BTC MOF nanotubes via a self‐sacrificing template strategy (Figure 4d).^105^ Briefly, Cd‐BTC MOF nanorods were first synthesized under ultrasonication in the presence of triethylamine (TEA). To convert those MOF nanorods to nanotubes, the subsequent reaction process was conducted without TEA (Figure 4e–h). In this work, TEA, which was served as the deprotonating agent, played an essential role in the morphology of prepared Cd‐BTC MOFs. In the presence of TEA, the kinetic growth process of MOF occurred, resulting in the formation of nanorods. On the other hand, the thermodynamically unstable MOF nanorods dissolved from inner part in the absence of TEA, leading to the formation of a nanotubular structure which is relatively stable.

#### 3.1.2 Template‐Free Method

Despite wide applicability of template method, tedious synthetic and removal procedures of templates impedes its large‐scale applications.^119^, ^120^, ^121^ A number of direct synthetic methods, namely template‐free method, toward 1D MOF structures have been developed so far, such as “rolling‐up” of 2D layer,^85^, ^97^, ^122^, ^123^, ^124^ crystal phase transformation,^52^, ^125^, ^126^ oriented assembly of designed organic ligands and metal ions,^92^, ^127^, ^128^ and so on. Taking “rolling‐up” method as an example, the following three factors, i.e., rationally designed geometry of SBUs, strong interlayer interaction and weak interlayer coupling of 2D layer, generally facilitate the morphology conversion from sheet to tube. In 2008, Li and co‐workers first reported the synthesis of 1D Cu‐MOF nanotube based on a “rolling‐up” process by a solvothermal reaction of CuCl_2_, 1,2,4‐Triazolate (Htz), and cyanuric acid (H_3_CA).^124^ In the synthetic process, the formed Cu_3_ SBUs have slightly curved planes, which were then curled up to form tubular nanostructure. Later in 2013, a sheet‐to‐tube conversion mechanism was proposed by Qiu and co‐workers, which regulated the host‐guest interactions among triethylamine and trinuclear Mn^II^_2_Mn^III^ units^123^ Based on this mechanism, a (4,0) zigzag metal‐organic nanotube was successfully achieved using Mn_3_(propandiolato)_2_ as the building block, and dicyanamide as the linker.

**Figure 5:**
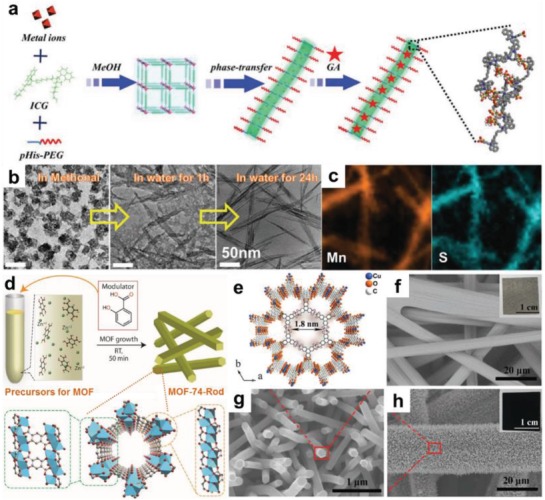
a) Schematic illustration of the preparation of Mn‐ICG@pHis‐PEG nanofibers. b) TEM images of Mn‐ICG@pHis‐PEG obtained in different solutions. c) STEM‐mappings of Mn‐ICG@pHis‐PEG nanofibers. Reproduced with permission.^52^ Copyright 2017, Wiley‐VCH. d) Scheme of the modulator‐assisted synthetic process of MOF‐74 rods. Reproduced with permission.^140^ Copyright 2016, Springer Nature. e) Crystal structure of a Cu‐CAT showing 1D channels along the c‐axis with open‐window size of 1.8 nm. SEM images of f) carbon fiber paper, and g,h) the Cu‐CAT nanowire arrays grown on a carbon fiber paper. Reproduced with permission.^89^ Copyright 2017, Wiley‐VCH.

Crystal phase transformation has been demonstrated to be another effective way to synthesize MOF‐based materials.^126^, ^129^, ^130^, ^131^, ^132^, ^133^, ^134^, ^135^, ^136^, ^137^ Previous studies have revealed that the phase transformation of MOFs can be achieved by substitution of metal ions and ligands, as well as removal of guest molecules within MOFs. Nevertheless, the preparation of 1D MOFs through crystal‐to‐crystal transformation was rarely reported. Recently, the conversion of 0D Cu coordination polyhedron (MOP‐1) to 1D Cu MOF was achieved by Wang and co‐workers through a link‐exchange strategy.^125^ Typically, MOP‐1 crystals were first synthesized through the solvothermal reaction of Cu^2+^ and 1,3‐benzenedicarboxylate. Then, the crystal conversion process was conducted by immersing MOP‐1 into 1‐methylimidazole, which partially occupied coordination sites of Cu^2+^, leading to unfolding of MOP‐1 and converting 0D to 1D structure. In addition to the aforementioned methods which introduced foreigner ligands, the direct phase transformation from bulk MOFs to 1D structures have been also achieved, which relies on properly adjusting the coordination between solvent and SBUs. For example, a series of 1D coordination polymers were synthesized by Liu and co‐workers through a rationally designed crystal phase transformation approach (**Figure** **5**a).^52^ In methanol phase, Mn^2+^, indocyanine green (ICG), and poly‐l‐histidine‐PEG (pHis‐PEG) are normally assembled to form Mn‐ICG@pHis‐PEG with a 3D structure, where Mn^2+^ is coordinated to 5 sulfonic acid groups from ICG and 1 imidazole from pHis‐PEG. While in water phase, the crystal phase transformation can be initiated, where H_2_O molecules with higher polarity plays the following two roles. (1) Water molecules competed with sulfonic acid groups from ICG to coordinate with Mn^2+^, and then formed a chain structure. (2) Adjacent chains were then connected by H_2_O molecules through hydrogen bonds, resulting in the formation of 1D Mn‐ICG@pHis‐PEG nanofibers (Figure 5b,c).

Moreover, adding coordination modulators, such as polyoxometalates,^138^ acetic acid,^26^, ^139^ and salicylic acid,^140^ is another simple and effective method to control the dimension and/or size of MOF crystals through directing nucleation and growth process of MOFs. For instance, Xu and co‐workers prepared rod‐shaped MOF‐74 with diameter of 30–60 nm and length of 200–500 nm using salicylic acid as a modulator (Figure 5d).^140^ As comparison, microcrystalline MOF‐74 was formed in the absence of salicylic acid. Similarly, ROD‐8 (Cd^II^),^92^ Co_5_(OH)_2_‐(O_2_CCH_3_)_8_·2H_2_O nanowires,^141^ NU‐1000 rods,^142^, ^143^ U‐based‐MOF nanotubes,^99^, ^144^ In‐porphyrin (Mn) hexagonal MOF rods,^53^ and Cu‐P,P′‐diphenyl‐diphosphinate (pcp)/1,2‐bis(4‐pyridyl)ethane) (bpye) nanorods^127^ were synthesized by directing their corresponding reactions of specifically designed organic ligands and metal ions. More recently, Xu and co‐workers fabricated conductive Cu‐CAT nanowire arrays on carbon paper by adding carbon paper into the reaction solution of Cu(Ac)_2_·H_2_O and 2,3,6,7,10,11‐hexahydroxytriphenylene (HHTP) (Figure 5e).^89^ By prolonging the reaction time for the Cu‐CAT nanowires, their length can be increased from 3 to 15 µm (Figure 5f–h).

Generally, template method is able to achieve well‐controlled structures for LD MOFs. The rationally designed templates along with their controlled transformation process allow the regulation of morphology and composition of 1D MOFs. It is also feasible to adjust the diameter and length of obtained 1D MOFs by choosing suitable templates. However, tedious processes including preparation and subsequent etching of template restrict the usage of template method for large‐scale synthesis of 1D MOFs. On the other hand, the direct synthesis of 1D MOFs, i.e., template‐free method, based on the reaction between specifically designed SBUs and organic ligands simplifies the synthetic process. Owing to the advantages of template‐free method, great progress has been achieved in recent years. Nevertheless, compared to rich synthetic chemistry of nanocrystals (e.g., CNTs, silicon nanowires, noble metal nanowires, nonprecious metal oxide nanorods, etc.),^38^, ^145^, ^146^ the direct synthesis of 1D MOFs is still in its infancy. It might be a future research direction for development of more effective and controllable methods to prepare 1D MOFs with certain compositions and structures. This could improve the performances of 1D MOFs in various applications as well as expand their usages in other new research fields.

### 3.2 Synthesis of 2D MOFs

**Figure 6:**
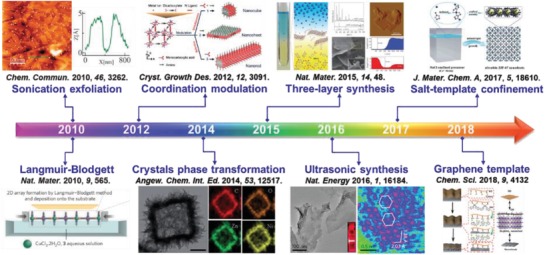
Timeline of important breakthroughs in the synthesis of 2D MOF nanosheets. Reproduced with permission.^147^ Copyright 2010, Royal Society of Chemistry. Reproduced with permission.^148^ Copyright 2010, Springer Nature. Reproduced with permission.^149^ Copyright 2012, American Chemical Society. Reproduced with permission.^150^ Copyright 2014, Wiley‐VCH. Reproduced with permission.^82^ Copyright 2015, Springer Nature. Reproduced with permission.^151^ Copyright 2016, Springer Nature. Reproduced with permission.^152^ Copyright 2017, Royal Society of Chemistry. Reproduced with permission.^153^ Copyright 2018, Royal Society of Chemistry.

**Figure 7:**
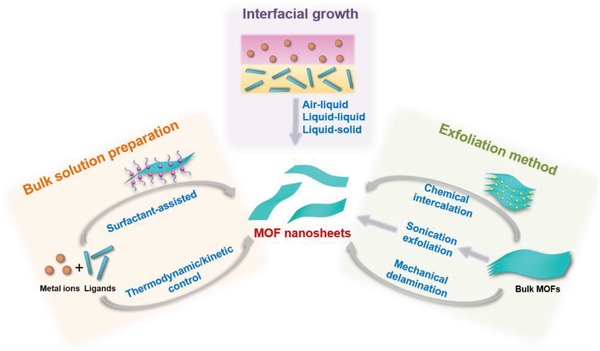
Schematic illustration of the three typical preparation strategies for 2D MOFs.

Preparation of ultrathin 2D MOFs with desired features, such as composition, size, thickness, crystal phase, defect, and surface property, are of particular importance, which enables further investigation of their physical, chemical, electronic, and optical properties, as well as exploration of various potential applications.^49^ Moreover, the compelling properties and promising applications of 2D MOFs promote rapid development of various reliable synthetic methods for 2D MOFs. **Figure** **6** exhibits those typical methods developed for the preparation of 2D MOFs in the past decade.^82^, ^147^, ^148^, ^149^, ^150^, ^151^, ^152^, ^153^ Generally, these methods can be classified into exfoliation, bulk solution preparation, and interfacial preparation. The synthetic procedures for these methods are schematically illustrated in **Figure** **7**.

#### 3.2.1 Exfoliation

**Figure 8:**
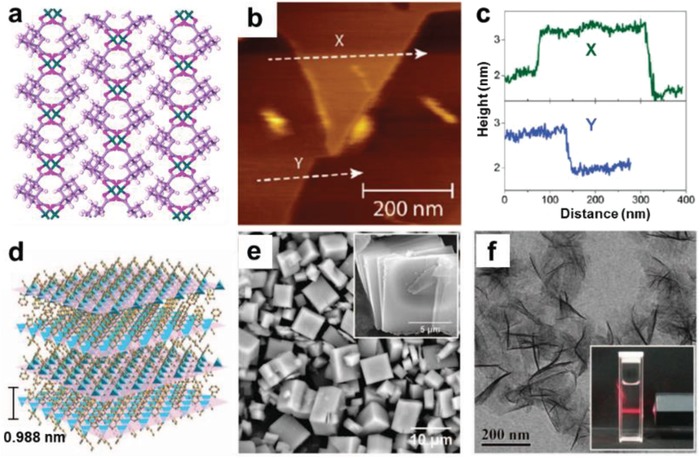
a) Crystal structure of Zn‐C_12_H_14_O_4_ MOF showing no chemical bond between its layers. Reproduced with permission.^158^ Copyright 2008, Royal Society of Chemistry. b) AFM image of the exfoliated Mn‐DMS nanosheet, and c) the corresponding height profiles. Reproduced with permission.^159^ Copyright 2011, American Chemical Society. d) Crystal structure of layered Zn_2_(bim)_4_ showing each Zn ion is coordinated by 4 benzimidazole ligands in a distorted tetrahedral geometry. e) SEM images of the bulk Zn_2_(bim)_4_ precursor. f) TEM image of Zn_2_(bim)_4_ nanosheets. Reproduced with permission.^160^ Copyright 2014, American Association for the Advancement of Science.

Layered bulk MOF crystals is able to be exfoliated into ultrathin 2D MOF nanosheets in liquid phase when proper mechanical forces are applied.^154^, ^155^, ^156^, ^157^ Sonication is one of the most common mechanical forces which can be simply applied for the exfoliation of layered bulk crystals to obtain ultrathin 2D MOF nanosheets. In 2008, Fjellvag and co‐workers first reported the delamination of Zn‐C_12_H_14_O_4_ MOF in acetone and ethanol.^158^ The crystallographic analysis proved the weak van der Waals bonding between the layers of this metal‐organic compound (**Figure** **8**a). After ultrasonication of Zn‐C_12_H_14_O_4_ MOF for ≈3 h at room temperature along with evaporation of acetone, delaminated single layers were observed. The thickness of particles is ≈2 nm, corresponding to two layers. Later on, Zamora and co‐workers reported weakening of the interlayer interactions within [Cu_2_Br(isonicotinato (IN))_2_]*_n_* to produce 2D nanosheets by a similar sonication process.^147^ Atomic Force Microscopy (AFM) topography image showed a homogenous distribution of nanosheets with thickness of ≈5 Å, which is in good agreement with the thickness of single‐layer [Cu_2_Br(IN)_2_]*_n_*. Cheetham and co‐workers also demonstrated the exfoliation of a layered hybrid framework material of Mn‐2,2‐dimethylsuccinate (DMS), in which layers are connected by weak van der Waals interactions.^159^ The exfoliated nanosheets display a thickness of ≈1 nm, which corresponds to the thickness of a single layer (Figure 8b,c). Apart from ethanol, the authors also attempted to use other solvents as the exfoliation agent, including water, methanol, hexane, and THF. However, ethanol was found to be the most efficient agent for exfoliating layered MOFs and preventing the isolated sheets from re‐stacking. Yang and co‐workers further revealed that wet‐ball‐milling approach facilitated the penetration of methanol molecules into the galleries of the layered Zn_2_(bim)_4_, and propanol benefited stabilization of the exfoliated nanosheets (Figure 8d–f).^160^ In addition, similar to the production of graphene by thermal expansion of graphite, a new strategy of thermal exfoliation has been reported on layered MOF crystals. Song and co‐workers demonstrated that layered metal‐hexamine (HMT) frameworks can be thermal exfoliated and simultaneously converted into 2D carbon‐based nanomaterials. During the rapid heating process, partial decomposition of HMT generated a large amount of gases (e.g., NO, NH_3_) to break the hydrogen bonds in the HMT interlayers, leading to the successful exfoliation.^161^ Recently, a chemical exfoliation approach by using chemical reactions to weaken the interlayer interactions has been developed to produce ultrathin MOF nanosheets with high yields.^156^

In general, it is a simple and feasible way to prepare 2D MOF nanosheets via exfoliation of layered bulk MOFs with assistance of external force, such as ultrasonication and ball‐milling. However, structural destruction and morphological distortion may simultaneously occur during these processes, which impede the preparation of high‐quality MOF nanosheets. In addition, lacking of precise regulation for the strength of applied external forces along with restacking problem of obtained MOF nanosheets make it difficult to control the thickness of MOF nanosheets. Besides, exfoliation method is generally applicable to layered MOFs with weak interlayer interaction. Therefore, extensibility of this method is limited. In order to realize the application of exfoliation method in practical industrial production, the aforementioned problems should be considered, and feasible solutions should be developed.

#### 3.2.2 Bulk Solution Preparation

**Figure 9:**
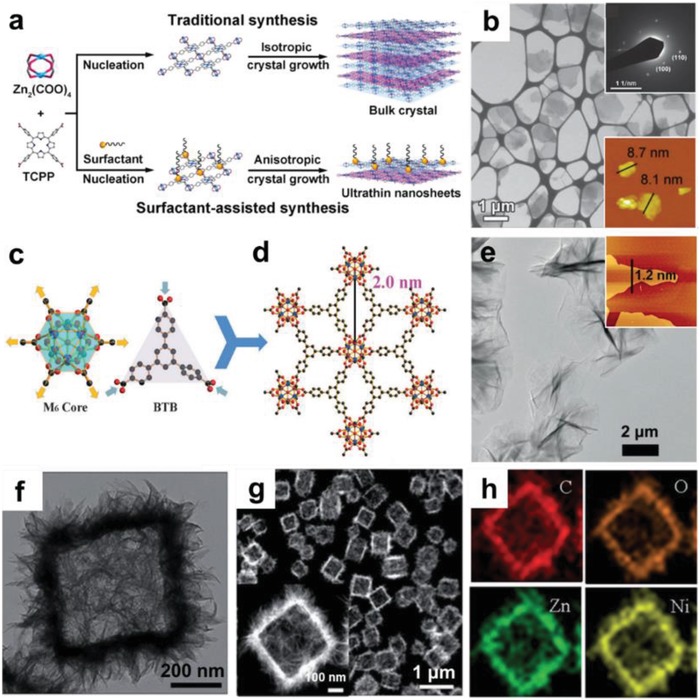
a) Schematic illustration of the traditional and surfactant‐assisted synthetic processes for TCPP‐based MOFs. b) TEM and AFM images of ultrathin Zn‐TCPP nanosheets. Reproduced with permission.^75^ Copyright 2015, Wiley‐VCH. c) Schematic illustrations of the Hf_6_ secondary building unit (SBU) (left), and the BTB ligand (right). d) Crystal structure and e) TEM image of Hf_6_O_4_(OH)_4_‐(HCO_2_)_6_(BTB)_2_ nanosheets. Reproduced with permission.^64^ Copyright 2016, Wiley‐VCH. f) TEM image, g) HAADF‐STEM image, and h) EDX elemental mappings of hierarchical Zn/Ni‐MOF‐2 nanosheet‐assembled hollow nanocubes. Reproduced with permission. Copyright 2014, Wiley‐VCH.^150^

Compared to exfoliation methods which generally have low yield and may cause morphological/structural destruction,^162^, ^163^, ^164^ direct synthesis of 2D MOFs in bulk solution is relatively effective in regulating the size and thickness of 2D nanosheets. The key of this method is to inhibit the growth of crystal in a certain direction.^48^, ^56^, ^84^ It is well known that surfactant is able to selectively attach to a certain crystallographic plane, which decreases surface energy and promotes anisotropic growth of crystal. This strategy has been also used for the preparation of MOF nanosheets^50^, ^165^, ^166^, ^167^ Zhang and co‐workers synthesized a series of tetrakis(4‐carboxyphenyl)porphyrin (TCPP) based ultrathin 2D MOFs through a surfactant‐assisted method, such as Zn‐TCPP, Cu‐TCPP, Cd‐TCPP, Co‐TCPP(Fe), Cu‐TCPP(Fe), Zn‐TCPP(Fe), and PPF‐3 nanosheets.^75^, ^168^, ^169^ As illustrated in **Figure** **9**a, bulk Zn‐TCPP crystals with layered structure were formed without addition of surfactants in the synthetic process.^75^ In each layer of bulk Zn‐TCPP, one Zn_2_(COO)_4_ paddlewheel metal node is coordinated with four TCPP ligands. While the introduced surfactant of polyvinylpyrrolidone (PVP) restricted the growth of Zn‐TCPP along the specific direction, leading to the formation of ultrathin Zn‐TCPP nanosheets with thickness of less than 10 nm (Figure 9b). Other surfactants, such as sodium lauryl sulfate^84^ and cetyltrimethyl ammonium bromide (CTAB)^170^ have also been demonstrated effective to the synthesis of MOF nanosheets.

In addition to surfactants, small capping molecules have also been used for the preparation of MOF nanosheets.^149^ The functional group of formate was used as modulator by Lin and co‐workers, which is able to reduce surface energy of crystals without blocking the active sites, and produce 2D MOF nanosheets.^78^, ^171^, ^172^, ^173^, ^174^ As a typical example, Lin and co‐workers used HCOO^−^ to protect six of the twelve connection sites of Hf_6_ cluster.^64^ The remaining six connection sites in the same plane are coordinated with organic ligand, i.e., benzene‐1,3,5‐tribenzoate (BTB), to form Hf_6_(µ_3_‐O)_4_(µ_3_‐OH)_4_‐(HCO_2_)_6_(BTB)_2_ MOLs (Figure 9c, d). Figure 9e shows the MOF nanosheets have a size of about 4 × 4 µm^2^ and a thickness of ≈1.2 nm, which is close to the size of Hf_6_ cluster. In another example, Wang and co‐workers synthesized ultrathin Zr‐TCPP(Ni) nanosheets with thickness of ≈1.48 nm through precisely controlling the concentration of modulator, i.e., monocarboxylic acids (formic acid, acetic acid, lauric acid, or oleic acid).^72^ The authors proposed a “pseudoassembly–disassembly” mechanism to illustrate the formation process of Zr‐TCPP(Ni) MOF nanosheets. The introduced modulator can occupy a portion of coordination sites of Zr_6_ SBUs, resulting in weakened interlayer interaction within the bulk MOF crystal. As the reaction time prolongs, the unstable bulk MOF begins to “disassemble” to minimize the surface energy, leading to the formation of 2D MOF sheets. Moreover, gluconate is used to coordinate with Zn ions and hinder the growth of Zn(bim)(OAc) MOF crystals in a certain direction, thereby achieving high yield preparation of ultrathin Zn(bim)(OAc) nanosheets with a thickness of about 5 nm.^175^

Using surfactants or small molecule modulators for the synthesis of 2D MOFs may block part of the active sites. In this regard, the synthetic methods without adding other molecules to achieve anisotropic growth of MOF nanosheets have received considerable attentions, such as the approaches via kinetic and/or thermodynamic controls over the growth process,^150^, ^176^ or using rationally designed ligands and SBUs with a predisposition to preferentially grow into a 2D structure.^87^, ^88^ As a typical example, the Zn/Ni MOF‐2 nanosheet‐assembled hollow structure was prepared through a surfactant‐free solvothermal approach.^150^ In this synthetic process, Zn/Ni‐MOF‐5 nanocubes were first formed under kinetic control. As the reaction time prolonged, the concentration of metal ions and ligands in the reaction system decreased, resulting in the formation of the thermodynamically favored Zn/Ni MOF‐2 nanosheets, as shown in Figure 9f–h.

The direct synthesis of 2D MOF nanosheets in bulk solution is capable of overcoming the shortcomings of the exfoliation method mentioned in Section 3.2.1, which produces high‐quality MOF nanosheets with tunable thickness. Relying on this synthetic strategy, a number of 2D MOF nanosheets have been successfully achieved, such as ultrathin Zr‐TCPP(Ni) nanosheets (thickness about 1.48 nm),^79^ Zr‐BPY nanosheets (thickness about 1.2 nm)^177^ and Hf‐4′‐(4‐benzoate)‐(2,2′,2′′‐terpyridine)‐5,5′′‐dicarboxylate) (TPY) nanosheets (thickness about 1.2 nm).^178^ Similar to other 2D materials, MOF nanosheets promote mass transport, which can effectively improve their performances in applications like catalysis and sensing. Moreover, ultrathin 2D MOF nanosheets with controlled thickness offer an ideal model system for the investigation of structural‐dependent performance at atomic/molecular levels. Nevertheless, the synthesis of 2D MOF nanosheets in bulk solution usually utilizes surfactants or modulators to confine the growth of MOF crystals in a certain direction. This inevitably blocks part of active sites of MOFs. Therefore, it is desirable to develop a suitable synthetic method which effectively controls the growth direction of crystal without using surfactant/modulator.

#### 3.2.3 Interfacial Growth

**Figure 10:**
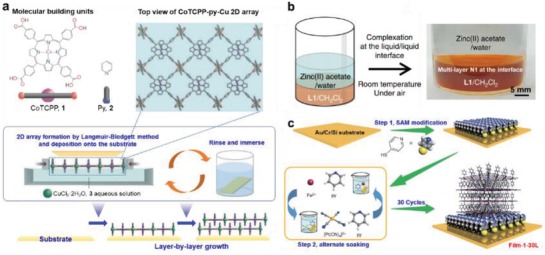
a) Schematic illustration of the fabrication procedure for TCPP(Co)‐pyridine‐Cu MOF nanofilms. Reproduced with permission.^148^ Copyright 2010, Springer Nature. b) Schematic illustration of the interfacial synthesis of bis(dipyrrinato)zinc(II) complex nanosheets at the interface of H_2_O and CH_2_Cl_2_. Reproduced with permission.^188^ Copyright 2015, Springer Nature. c) Schematic illustration of the fabrication process for Fe(pyridine)_2_[Pt(CN)_4_] thin film at liquid/solid interface. Reproduced with permission.^193^ Copyright 2016, Springer Nature.

It is an effective method for preparing MOF nanosheets by confining the growth of MOF crystals at an interface between two different phases.^179^, ^180^, ^181^, ^182^, ^183^, ^184^ In the following paragraphs, we will briefly introduce the synthesis of MOF nanosheets at various interfaces, including air–liquid, liquid–liquid, and solid–liquid interfaces. As a typical example for air–liquid interfacial synthesis of 2D MOFs, Kitagawa and co‐workers reported the fabrication of TCPP(Co)‐pyridine‐Cu MOF nanofilms (NAFS‐1) through a Langmuir–Blodgett approach and the subsequent layer‐by‐layer (LbL) growth (LB‐LbL).^148^ The ligands of TCPP(Co) and pyridine pillaring molecules were first dissolved in a mixed solvent containing chloroform and methanol, and then spread onto an aqueous solution of copper chloride to form TCPP(Co)‐pyridine‐Cu sheets on the air–liquid interface (**Figure** **10**a). Later on, the same group reported the fabrication of TCPP‐Cu nanofilm (NAFS‐2) on Au and Si surfaces through a similar LB‐LbL method.^185^ Similarly, single‐layer TCPP(Pd)‐Cu MOF nanosheets,^186^ Cobalt dithiolene films,^187^ and single‐/few‐layer nickel bis(dithiolene) nanosheets were fabricated at air–liquid interfaces.

The liquid–liquid interface formed by two immiscible solvents restricts the isotropic growth of crystals, and produces 2D MOFs. For instance, Nishihara and co‐workers reported the fabrication of multilayer bis(dipyrrinato)zinc complex nanosheets at H_2_O/CH_2_Cl_2_ interface (Figure 10b).^188^ Specifically, adding the aqueous solution of Zn(Ac)_2_ to the CH_2_Cl_2_ solution with dipyrrin ligand formed a water/oil interface, in which Zn^2+^ and dipyrrin ligands spontaneously assembled at the interface and react to form 2D MOF films. Furthermore, it was demonstrated that the thickness of MOF film can be controlled by adjusting the concentration of dipyrrin ligand in CH_2_Cl_2_. Recently, the same group prepared a bis‐(terpyridine)‐zinc(II)^189^ complex at the H_2_O/CH_2_Cl_2_ interface relying on a similar procedure, demonstrating the versatility of this method.^190^, ^191^, ^192^ Additionally, 2D MOFs can be also prepared at liquid–solid interfaces. For example, Fe(pyridine)_2_[Pt(CN)_4_] thin film was prepared by LbL growth on a Au/Cr/Si substrate (Figure 10c).^193^ In this process, a substrate modified by pyridine‐terminated thiol(4‐mercaptopyridine) was alternately immersed in two solutions of Fe^2+^ and [Pt(CN)_4_]^2−^, respectively, to form the 2D MOF film. Other 2D MOF films, such as Fe[Pt(CN)_4_],^194^ Fe(pyridine)_2_Ni(CN)_4_,^195^ and Fe(pyrazine)[Pt(CN)_4_],^196^ have also been prepared at the liquid–solid interface through LbL method.

#### 3.2.4 Other Synthetic Methods

In addition to the aforementioned methods, synthesis of the 2D MOF nanosheets can be also achieved through other approaches.^197^, ^198^ As a typical example, Dong and co‐workers reported the preparation of ultrathin ZIF‐67 nanosheets through a salt‐template confinement method.^152^ The solution that presented in the small gaps among NaCl microcrystals served as the precursors for ZIF‐67 crystal, leading to growth of 2D nanosheets along the microcrystal plane of NaCl. Recently, some 2D nanomaterials, such as GO sheets,^153^ and Cu(OH)_2_ nanobelts^83^ were used as template to guide the growth of 2D MOF nanosheets. Besides, pseudoassembly–disassembly strategy^79^ and chemically selective cleavage strategy^199^ have also been developed to prepare 2D MOF nanosheets.

### 3.3 Synthesis of LD MOF‐Based Composites

Integration of MOF with other functional materials, such as other types of MOFs,^200^, ^201^, ^202^ metal oxides,^203^, ^204^ metal NPs,^205^, ^206^, ^207^ polyoxometalates,^208^, ^209^ covalent‐organic frameworks (COFs),^210^, ^211^, ^212^ endows the resultant MOF‐based composites with improved physical and chemical properties as well as new functions.^213^ Especially, previously reported LD MOF‐based composites normally exhibited enhanced performances, which are arised from the successful combination of advantages of both LD MOFs and other functional materials. For example, enhanced photovoltaic performance in photoelectrochemical aplication can be achieved after the integration of the advantages of high flexibility and specific surface area from LD MOFs with the unique exciton separation property from semiconductor NPs.^214^ To date, a variety of rationally designed LD MOF‐based composites, such as MIL‐88 rod@ZIF‐8 composite,^215^ Fe‐MOF NP/ Ni‐MOF nanosheet composite,^216^ and Au NP/Cu‐TCPP(Fe) nanosheet composite,^59^ have been prepared via various methods, such as in‐situ reduction method, epitaxial growth method, etc. In this section, we summarize 1D and 2D MOF‐based composites, including the LD MOF‐based composites with other type of MOFs and non‐MOF components, with emphasis on their preparation methods.

#### 3.3.1 LD MOF/MOF Composites

The proper combination of two different MOFs provides great opportunities to overcome their inherent disadvantages, create new functions, and improve properties, which has become an effective way to construct multifunctional MOF composites. For example, size‐selective catalysis can be achieved by coating a ZIF‐8 layer with molecular sieving behavior on the surface of a presynthesized MOF core with catalytic activity. The preparation of MOF‐MOF composites by heteroepitaxial growth or postsynthetic modification (PSM) has been extensively studied in recent years, although most of reports are limited to bulk MOF crystals.^213^ For instance, Zhou and co‐workers reported a core‐shell structured PCN‐134@Zr‐BTB composite with internal and external diameters of ≈30 and 80 µm, respectively.^217^ Li and co‐workers prepared a core‐shell ZIF‐67@ZIF‐8 polyhedron with an external diameter of ≈500 nm through a seed‐mediated growth technique.^218^ On the contrary, the research on LD MOF/MOF composites with sophisticated architectures based on 1D and/or 2D MOF matrix, is still in its infancy.^219^

**Figure 11:**
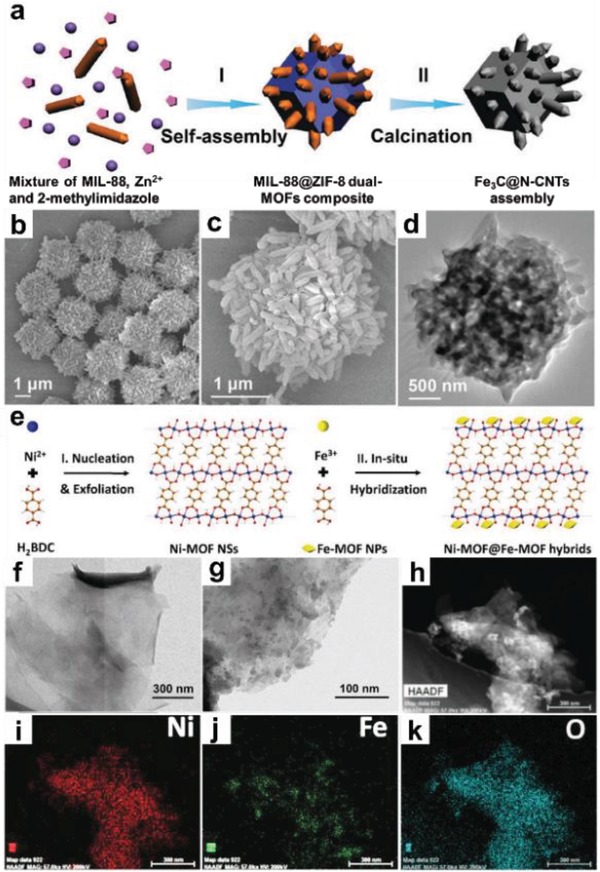
a) Schematic illustration of the synthetic process for MIL‐88@ZIF‐8 composite and its derivative. b,c) FESEM and d) TEM images of MIL‐88@ZIF‐8 composites. Reproduced with permission.^215^ Copyright 2016, Royal Society of Chemistry. e) Schematic illustration of the preparation process for Ni‐MOF/Fe‐MOF composite nanosheet. TEM images of f) Ni‐MOF and g) Ni‐MOF/Fe‐MOF composite nanosheet. h–k) HAADF‐STEM image and corresponding elemental mappings of Ni‐MOF@Fe‐MOF hybrid nanosheets. Reproduced with permission.^216^ Copyright 2018, Wiley‐VCH.

Recently, Lou and co‐workers reported a novel MOF‐in‐MOF structure, which was prepared by reacting presynthesized Fe‐based MIL‐88 crystals with Zn^2+^ ions and 2‐methylimidazole (**Figure** **11**a).^215^ As shown in Figure 11b–d, MIL‐88 nanorods are uniformly anchored on ZIF‐8 polyhedrons. It was demonstrated that the introduction of MIL‐88 nanorods into the ZIF‐8 host guaranteed the uniform distribution of Fe_3_C nanocrystals within its pyrolyzed product. In addition, the ZIF‐8 matrix was converted to porous N‐doped carbon species which protect Fe_3_C nanocrystals from agglomeration. The authors also demonstrated that these hierarchically structured materials exhibited excellent electrocatalytic activity in oxygen reduction reaction (ORR).^215^ Later on, MoO_3_@MIL‐88/ZIF‐67 hybrid rods and GO@MIL‐88/ZIF‐67 hybrid sheets with unique “MOF‐in‐MOF hybrid” structure were demonstrated via similar self‐assembly methods by the same group.^220^, ^221^ Recently, the Fe‐MOF NP‐coated Ni‐MOF nanosheet (Fe‐MOF/Ni‐MOF composite) was obtained by a stepwise conversion method (Figure 11e).^216^ Ni‐MOF nanosheets with thickness of ≈5 nm were first prepared by ultrasonication of BDC, NiCl_2_ and triethylamine in a mixed solvent of DMF, ethanol, and water (Figure 11f). Subsequently, the as‐obtained Ni‐MOF nanosheets were added to another mixture containing Fe^3+^ and BDC. Under ultrasonication, metal ions and organic ligands were anchored onto Ni‐MOF nanosheets and in situ coordinated to form Fe‐MOF NPs, which resulted in the formation of Fe‐MOF/Ni‐MOF composite nanosheets (Figure 11g–k).^216^

#### 3.3.2 LD MOF/Non‐MOF Composites

In recent years, the integration of 1D and/or 2D MOFs with a variety of functional materials, such as metal oxides, metal NPs, and COFs, has been extensively studied, which combines the merits from all components and eliminates the shortcoming of individual component. To date, many LD MOF/non‐MOF composites have been synthesized, such as cellulose@ZIF‐67 nanowires,^111^ Ru/Zn‐BTC fibers,^222^ ZnO@ZIF‐8 rods,^223^, ^224^ Cu(HBTC)‐1/Fe_3_O_4_‐AuNPs nanosheets^225^ and so on.

**Figure 12:**
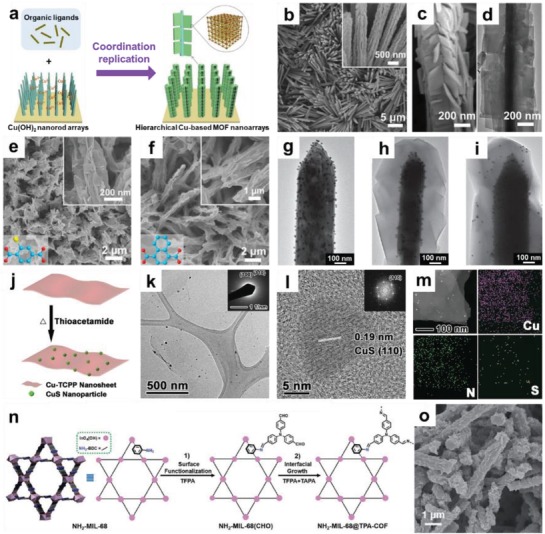
a) Schematic illustration of synthetic process for hierarchical Cu‐based MOF nanoarrays. b,c) SEM and d) TEM images of hierarchical MOF‐2 nanoarrays. SEM images of e) Cu‐based MOF nanoarrays with different ligands of Br‐H_2_BDC, and f) 1,4‐H_2_NDC. Reproduced with permission.^226^ Copyright 2017, Wiley‐VCH. TEM images of g) ZnO nanorods@Au, and h,i) ZnO nanorods@Au@ZIF‐8 composites with Au NPs at different locations. Reproduced with permission.^116^ Copyright 2017, Springer Nature. j) Schematic illustration showing the preparation process for the CuS/Cu‐TCPP composite nanosheet. k) TEM and l) HRTEM image of the CuS/Cu‐TCPP composite nanosheets. m) Dark‐field STEM image of CuS/Cu‐TCPP composite nanosheets and the corresponding elemental mapping. Reproduced with permission.^214^ Copyright 2016, Wiley‐VCH. n) Schematic illustration of the synthetic process and o) SEM image of hierarchical NH_2_‐MIL‐68@TPA‐COF composite. Reproduced with permission.^211^ Copyright 2017, Wiley‐VCH.

The synthetic strategy for MOF/non‐MOF composites can be divided into two categories: 1) as‐synthesized nanomaterials are served as templates for the subsequent nucleation and growth of MOFs; 2) direct growth of other functional nanomaterials on presynthesized MOF crystals with certain morphology and structure. For example, Liu and co‐workers developed a coordination replication method for the preparation of various hierarchical structures of Cu(OH)_2_ nanorod@Cu‐based MOF nanosheet (**Figure** **12**a).^226^ The as‐synthesized Cu(OH)_2_ nanorods were first added into a benzyl alcohol solution of H_2_BDC, in which Cu(OH)_2_ was etched to gradually release Cu^2+^. Subsequently, the deprotonated organic ligand was coordinated in situ with Cu^2+^ to form MOF‐2 nanosheets which vertically grown on Cu(OH)_2_ nanorods (Figure 12b–d). The authors also synthesized other Cu(OH)_2_ nanorod@MOF nanosheet hierarchical structures based on this strategy. By changing the organic ligands to Br‐H_2_BDC and 1,4‐H_2_NDC, Cu(OH)_2_ nanorod@Cu(Br‐BDC) nanosheet arrays (Figure 12e) and Cu(OH)_2_ nanorod@Cu(1,4‐NDC) nanosheet arrays (Figure 12f) were obtained, respectively. More recently, the same group prepared ZnO@Au@ZIF‐8 nanorods through a similar template‐sacrifice method using ZnO nanorod@Au NPs as the template.^116^ Interestingly, the authors revealed that the nucleation‐growth process of MOFs can be regulated by tuning the concentration of organic ligands. Specifically, when ZnO nanorod@Au NPs template was added into a solution with high concentration of ligand, a large number of Zn^2+^ ions were rapidly released to the bulk solution (Figure 12g). This promotes the nucleation of ZIF‐8 in the solution followed by deposition on the surface of ZnO nanorod, and the Au NPs were at the interface between ZnO and MOF layers (Figure 12h). On the contrary, when ZnO nanorod@Au NPs was added into a solution with low concentration of ligand, only trace amount of Zn^2+^ ions diffused into the solution around ZnO nanorod@Au NPs making the homogeneously nucleation of ZIF‐8 in solution difficult. In this case, ZIF‐8 nucleated and grew on the surface of ZnO, which forced the migration of Au NPs from the surface of ZnO nanorod to that of ZIF‐8 (Figure 12i).^116^

The in situ growth of other functional materials on presynthesized 1D and/or 2D MOFs is another feasible way to obtain LD MOF composites. For example, CuS/Cu‐TCPP composite nanosheet were prepared by in situ sulfidation of corresponding MOF matrix.^214^ As illustrated in Figure 12j, Cu‐TCPP nanosheets were first synthesized by a surfactant‐assisted synthetic method. It was then dispersed into an ethanol solution containing thioacetamide, and heated to 75 °C for 3 h. During this process, Cu‐TCPP nanosheets were partially sulfurized to form CuS NPs (Figure 12k–m). Based on a similar approach, CdS/Cd‐TCPP and CoS*_x_*/Co‐TCPP composite nanosheets were prepared. Recently, Au NP/Cu‐TCPP(Fe) and Au NP/Cu‐TCPP(Co) composite nanosheets were prepared by reduction of HAuCl_4_ with NaBH_4_ in the presence of corresponding MOF nanosheets.^59^ In addition to hybridizing MOFs with inorganic nanomaterials, the construction of MOF/organic material composites has also been demonstrated.^227^ For example, Zhang and co‐workers reported a strategy for controlled synthesis of MOF@COF core‐shell hybrid material.^211^ First, NH_2_‐MIL‐68 rods were synthesized by a solvothermal method, and then converted into NH_2_‐MIL‐68(CHO) through surface functionalization with tris(4‐formylphenyl)amine (TFPA) (step 1 in Figure 12n). After that, NH_2_‐MIL‐68@TPA‐COF composite was obtained by condensing TFPA and tris(4‐aminophenyl)amine (TAPA) on NH_2_‐MIL‐68(CHO) rods (step 2 in Figure 12n). As shown in Figure 12o, TPA‐COF sheets with thickness of 50–200 nm were uniformly coated on NH_2_‐MIL‐68 rods, forming a MOF@COF core‐shell hierarchical structure.

### 3.4 Synthesis of LD MOF Derivatives

**Figure 13:**
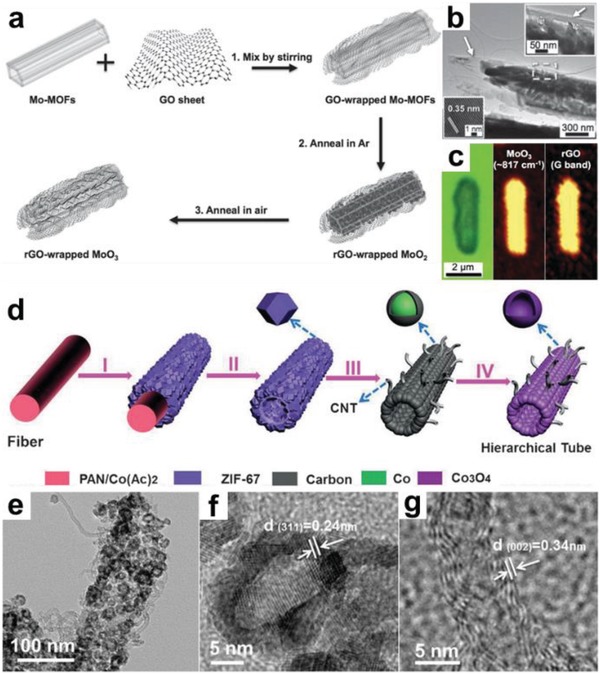
a) Schematic illustration of synthetic process for the rod‐like rGO/MoO_3_ composite. b) TEM image and c) Raman mappings of the rGO/MoO_3_ composite. Reproduced with permission.^73^ Copyright 2015, Wiley‐VCH. d) Schematic illustration of the preparation of hierarchical CNT/Co_3_O_4_ microtubes. e–g) TEM images of the hierarchical CNT/Co_3_O_4_ microtubes. Reproduced with permission.^244^ Copyright 2016, Wiley‐VCH.

Recent works have clearly demonstrated MOF‐based materials are excellent sacrificial templates or precursors for the preparation of a variety of functional materials, such as metal‐based compounds, porous carbon materials, and their composites, through pyrolysis under specific atmospheres (e.g., Ar, N_2_, air) or reaction with certain reagents.^221^, ^228^, ^229^ This section describes the synthetic strategies of LD MOF‐derived materials. Morphology‐preserved pyrolysis is a widely used synthetic method for 1D MOF derivatives by using rod‐like or wire‐like MOFs as precursors/templates under a proper condition which preserve original 1D morphology. As a typical example, porous Co_3_O_4_ rods were prepared via direct annealing of rod‐like Co‐MOF‐74 precursors in air.^230^ Similarly, mesoporous Ni*_x_*Co_3−_ *_x_*O_4_ rods were obtained by calcination of Co/Ni‐MOF‐74 rods at 450 °C under air atmosphere.^72^ Interestingly, 1D porous metal oxide/carbon composites (e.g., MnO/C, Co_3_O_4_/C) were obtained by changing the calcination atmosphere to N_2_ 231, 232 or Ar.^233^ 1D MOF‐derived porous carbon structures can be also achieved after removing metallic species by evaporation at high temperatures^140^ or acid (HF, H_2_SO_4_, etc.) etching.^234^, ^235^, ^236^ Recently, Co*_x_*Ni*_y_*P nanotubes with various Co/Ni ratios were synthesized via a two‐step calcination method.^237^ In this process, Co*_x_*Ni*_y_*O was first obtained by calcining Co/Ni‐MOF‐74 rods in air, followed by a thermal treatment with NaH_2_PO_2_ in nitrogen atmosphere to produce Co*_x_*Ni*_y_*P nanotubes. To improve the electrical conductivity and structural stability of MOF derivatives, we developed a preparation method for GO‐wrapped Mo‐MOF (GO/Mo‐MOF) rod through a simple mixing process of Mo‐MOF and GO nanosheet. The obtained GO/Mo‐MOF was then used as precursor/template for reduced graphene oxide‐wrapped MoO_3_ (rGO/MoO_3_) (**Figure** **13**a–c).^73^ LD MOF derivatives can be also obtained by deposition of MOFs on certain substrates, which not only avoids agglomeration of the generated active components during calcination, but also endows MOF derivatives with new functions.^112^, ^142^, ^222^, ^238^, ^239^, ^240^ For example, TiO_2_ nanorods have been used as substrate for the growth of Cu‐BTC MOF‐coated TiO_2_, which severed as the precursor for the subsequent preparation of CuO NP‐coated TiO_2_ nanorods.^238^ Up to now, a variety of 1D structured nanomaterials, e.g., Te nanowire, ZnO, MnO_2_, CoO, and MIL‐88 rods, have been used as substrates for the preparation of LD MOF derivatives, which include porous carbon fibers,^112^ Te@ZnCo_2_O_4_ nanofibers,^113^ CoO@S‐Co_3_O_4_ nanorod arrays,^239^ NiFe_2_O_4_/Fe_2_O_3_ nanotubes,^240^ and heteroatom‐doped carbon materials with rod‐like morphologies.^65^, ^114^, ^241^, ^242^, ^243^ Recently, Lou and co‐workers used polyacrylonitrile (PAN) as sacrificial template to synthesize CNTs/Co_3_O_4_ microtubes through a multistep approach, which includes electrospinning, selective dissolution and calcination processes (Figure 13d).^244^ TEM images reveals the microtubes are mainly composed of hollow Co_3_O_4_ NPs with size of 10–20 nm and CNTs with inner diameter of about 3 nm (Figure 13e–g).

**Figure 14:**
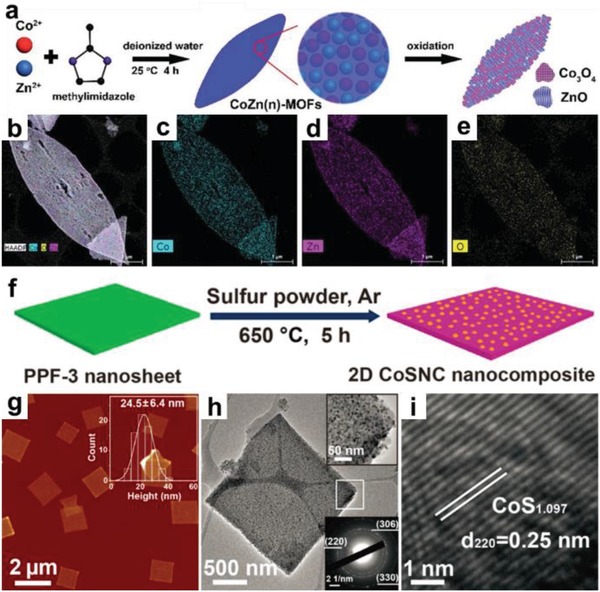
a) Schematic illustration of the preparation of 2D porous Co_3_O_4_/ZnO hybrid nanosheets. b–e) Elemental mappings of the Co_3_O_4_/ZnO nanosheets. Reproduced with permission.^248^ Copyright 2017, Royal Society of Chemistry. f) Schematic illustration of the formation process for 2D CoS_1.097_ NPs/nitrogen‐doped carbon (CoSNC) nanosheets. g) AFM, h) TEM, and i) HRTEM images of 2D CoSNC nanosheets. Reproduced with permission.^169^ Copyright 2016, American Chemical Society.

As compared to 1D MOF derivatives, the research on 2D MOF‐derived materials is in its infancy, which is mainly due to the challenges for high‐yield production of MOF nanosheets, and preventing their agglomeration during calcination.^152^, ^175^, ^245^, ^246^, ^247^ To solve these problems, salt‐template confined strategy has been developed by Dong and co‐workers, which involves the confined assembly of Co^2+^ and 2‐methylimidazole in the gaps among the salt microcrystals to form ultrathin ZIF‐67 nanosheets, which were served as precursors for porous Co, N co‐doped carbon nanosheets.^152^ Bimetallic MOF nanosheets have been proven to be ideal precursors for the synthesis of binary metal oxide nanosheets. Typically, Co_3_O_4_/ZnO nanosheet obtained by calcination of bimetallic 2D CoZn‐ZIF, showed uniform dispersion of Co and Zn on the bimetallic oxide nanosheets (**Figure** **14**a–e).^248^ In another work, Co NP/nitrogen‐doped CNTs were produced after calcination of CoZn‐ZIF nanosheets at 900 °C for 2h under Ar.^71^ Recently, the rationally designed metal coordination complexes, such as Fe‐1,4‐bis(1H‐1,3,7,8‐tetraazacyclopenta (1) phenanthren‐2‐yl)benzene (btcpb) nanosheets,^249^ Cu(4,4′‐bipy)(NO_3_)_2_,^250^ and Mo‐polydopamine nanopetals,^84^ have been served as precursors for the preparation of 2D metal‐nitrogen doped carbon materials. In addition to the aforementioned methods using metal oxides or carbon‐based complexes as 2D templates and/or precursors for 2D MOF derivatives, an interesting synthetic method was developed, which combines the chemical vapor deposition (CVD) of specific reagents with the pyrolysis of MOFs. For instance, CoS_1.097_ NPs/nitrogen‐doped carbon (CoSNC) nanosheets were obtained by simultaneous sulfidation and carbonization of 2D porphyrin paddlewheel framework‐3 (PPF‐3) (Figure 14f).^169^ The AFM measurement and TEM images confirm the CoSNC nanosheets with thickness of 24.5 ± 6.4 nm consisting of CoS_1.097_ NPs and carbon skeleton (Figure 14 g–i).

In order to alleviate the problems of agglomeration and structure collapse of 2D MOF during calcination, it is an effective way to grow MOF crystals directly on conductive substrates (e.g., carbon cloth, nickel foam, etc.) to construct MOF arrays.^251^, ^252^, ^253^, ^254^ Up to now, various 2D MOF‐derived nanostructure arrays have been reported, such as Ni‐doped Co‐Co_2_N nanosheet/carbon cloth,^255^ N‐doped carbon‐Co_3_O_4_ sheet/carbon cloth,^256^ Co_3_O_4_@N‐doped carbon nanosheet/nickel foam,^257^ and CoSe_2_ sheets/carbon cloth.^258^ Besides, multistep pretreatments before calcination, such as anion exchange and etching, can obtain MOF derivatives with more complex composition and/or structure.^61^ For example, Wang and co‐workers reported a two‐step strategy, i.e., ion exchange and etching process followed by thermal treatment in air, to prepare hollow NiCo_2_O_4_ nanowall arrays on carbon cloth.^259^ Furthermore, 2D MOF‐derivatives can be also obtained through hydrothermal/solvothermal reaction. A porous zinc cobalt sulfide nanosheet array grown on Ni foam was prepared in a simple sulfurization reaction with thioacetamide, which was based on the sacrificial template of 2D Zn/Co‐MOF nanosheets.^260^ Recently, a novel one‐step and energy‐saving method was proposed to synthesize 2D MOF‐derivatives. Specifically, Co‐MOF nanosheet arrays was converted to hollow Co(VO_3_)_2_‐Co(OH)_2_ composite leaf arrays at room temperature through exchange reactions between VO_3_^−^/OH^−^ and 2‐MIM ligands in Co‐MOF.^260^

## 4 Applications

LD MOF‐based materials have received dramatic attention in various research fields owing to the successful combination of the unique features of LD materials including high aspect ratio and abundant accessible active sites, as well as structural and crystalline characteristics of MOFs, such as diverse pore structure and high surface area.^1^, ^3^, ^9^, ^261^, ^262^, ^263^, ^264^, ^265^, ^266^, ^267^, ^268^, ^269^ Furthermore, LD MOF can be acted as versatile precursors or sacrificial templates to prepare numerous functional nanomaterials for various applications. The simple synthesis of MOFs provides a facile approach to obtain MOF‐derivatives with structural complexity and tunable composition. Owing to the regular arrangement of metal ions and organic ligands in MOF crystals, the obtained MOF‐derivatives usually have uniform distribution of different components (e.g., metal nanoparticles and carbon) with nanoporous structures. Moreover, organic ligands of MOFs enable the formation of carbon‐based nanomaterials after calcination, leading to the improvement of the electrical conductivity and electrochemical activity of MOF‐derivatives.^228^, ^270^, ^271^, ^272^ In this section, we highlight some promising applications of LD MOF‐based materials as well as their derivatives, including catalysis, gas adsorption and separation, energy storage, sensing, etc.

### 4.1 Catalysis

#### 4.1.1 Electrocatalysis

**Figure 15:**
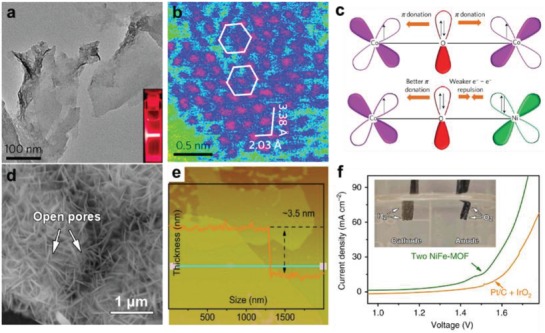
a) TEM image of ultrathin Ni/Co MOF nanosheets. The inset shows the Tyndall light scattering of composite solution. b) HAADF‐STEM image of the (200) plane of ultrathin Ni/Co MOF nanosheets, which shows hexagonal crystal lattice for the metal atoms (pink color). Among them, the green color stands for background and the blue represents carbon and oxygen. c) Schematic illustration of the electronic coupling between Co and Ni in NiCo‐UMOFNs. Reproduced with permission.^151^ Copyright 2016, Springer Nature. d) SEM image, and e) AFM image with corresponding height profile of NiFe‐MOF nanosheets. f) LSV plots of the electrocatalytic water splitting cell based on NiFe‐MOF electrodes, and the cell using a Pt/C cathode and a IrO_2_ anode. Reproduced with permission.^278^ Copyright 2017, Springer Nature.

Although LD MOFs possess high specific surface area and abundant intrinsic metal sites, the poor conductivity and low mass permeability limit their application in electrocatalysis.^273^ Recent work revealed that electrocatalytic performances of MOFs in oxygen evolution reaction (OER), hydrogen evolution reaction (HER) and oxygen reduction reaction (ORR) can be realized by electrically conductive MOFs and MOF derivatives (**Table** **2**).^3^, ^274^, ^275^, ^276^, ^277^ Tang and co‐workers prepared ultrathin Ni/Co MOF nanosheets (NiCo‐UMOFNs) with thickness of ≈3.1 nm via a simple ultrasonication method, and then used as electrocatalyst for OER in alkaline conditions.^151^ TEM image in **Figure** **15**a shows that the edges of the nanosheets are curled due to their ultrathin morphology. HAADF‐STEM image obtained in the low‐voltage mode (80 kV) further reveals the ordered distribution of metal atoms in the 2D bimetallic MOF nanosheet (Figure 15b). OER performance of NiCo‐UMOFNs is superior to that of their bulk counterpart, due to more accessible active sites. Moreover, the authors proposed that the coordinatively unsaturated metal sites and the coupling effect of Co and Ni species in bimetallic MOF nanosheets are responsible for their high electrocatalytic activity in OER (Figure 15c). Lang and co‐workers also reported that the synergistic effect of the metal species, i.e., Fe, Ni, and Co, can improve OER performance of Fe/Ni/Co‐MIL‐53 rods.^90^ The catalyst with optimized components, i.e., Fe/Ni_2.4_/Co_0.4_‐MIL‐53 rods, required a small overpotential of 219 mV to gain a current density of 10 mA cm^−2^, which is much lower than that of other Fe/Ni‐based MIL‐53, such as Fe/Ni*_x_*‐MIL‐53 (*x* = 1.6, 2.0, 2.4), Fe/Ni/Co_0.2_‐MIL‐53 and Fe/Ni_2.4_/Mn_0.2_‐MIL‐53 rods. Recently, Dou and co‐workers reported the introduction of Fe‐MOF NPs onto Ni‐MOF nanosheets to form Fe‐MOF/Ni‐MOF composite, which exhibited dramatically improved OER performance.^216^ In addition to the above examples, pristine LD MOFs and their composites can be also converted to active materials for OER via heat treatment, such as metal oxides, metal phosphates, and porous carbon‐based materials. For instance, porous CoSe_2_ nanosheet arrays derived from Co‐MOF nanowalls exhibited an overpotential of 290 mV at a current density of 10 mA/cm^2^, which is much lower than that of Co‐MOF nanowalls.^258^ Similarly, Co_3_O_4_/C nanowire arrays^231^, ^233^ and hollow NiCo_2_O_4_ nanostructure^259^ have been also prepared via thermally conversion of their corresponding LD MOF precursors, and then used as efficient OER electrocatalysts.

**Table 2:** Electrocatalytic activities of some typical LD MOF‐based and LD MOF‐derived electrocatalysts for OER, ORR, and HER

Nanomaterials	Structure	Target reactions	η_10_ or *E*_half‐wave_ [mV vs RHE]	Tafel slope [mV dec^−1^]	Stability	Ref.
NiCo‐MOF	2D	OER	189	42	200 h	151
	Bulk		317	61	/	
NiFe‐MOF	2D	OER	240	34	20 000 s	278
	Bulk		318	56	/	
Co‐MOF derived layered Co/N/C	2D	HER	103	/	2000 cycles	246
THTA‐Co MOF	2D	HER	283	71	300 cycles	279
MOF‐74 derived Co/Ni‐P	1D	OER	245	61	20 h	237
		HER	129	52	3000 cycles	
ZIF‐67 derived Ni@CoO@Co/N/C	1D	HER	190	98	20 h	91
		OER	309	53	20 h	
Co‐MOF derived Co_3_O_4_/C nanowire arrays	1D	OER	η_30_: 318	81	1000 cycles	233
ZIF‐67/Ti@TiO_2_/CdS	2D	OER	410	42	30 h	277
Co‐MOF derived CoSe_2_	2D	OER	290	114.7	24 h	258
Co‐MOF derived hollow NiCo_2_O_4_ nanowall arrays	2D	OER	340	72	20 h	259
Co‐based MOF derived Co_3_O_4_/C	1D	OER	290	70	30 h	231
		ORR	780	89	30 h	
Fe/Ni_2.4_/Mn_0.4_‐MIL‐53	1D	OER	η_20_: 236	52.2	60 h	90
Ni‐MOF@Fe‐MOF	2D	OER	265	82	1000 cycles	216
Co/Zn MOF derived Co‐N‐CNTs	1D/2D	OER	460	/	10 000 cycles	71
		ORR	900	/	10 000 cycles	
Fe‐btcpb derived Fe−N/C	2D	ORR	840	/	8000 cycles	249
ZIF‐8 derived porous carbon nanofibers	1D	ORR	−161 (vs Ag/AgCl)	/	8000 cycles	112
ZIF‐67 derived Co,N‐C	2D	ORR	869	50.5	36 000 s	152

LD MOF‐based materials and their derivatives have also been investigated as electrocatalyst for HER.^187^ As a typical example, Zhao and co‐workers prepared ultrathin Ni/Fe MOF nanosheet arrays via a chemical‐bath‐deposition method.^278^ 2D Ni/Fe MOF nanosheets with thickness of ≈3.5 nm were grown vertically on the nickel foam, forming a large number of open pores (Figure 15d,e). The Ni/Fe‐MOF nanosheets exhibited high electrocatalytic activity for HER with a low overpotential of 134 mV at the current density of 10 mA cm^−2^. Remarkably, the chronoamperometry tests indicate the excellent durability of Ni/Fe‐MOF for HER over 2000 s. Furthermore, the ultrathin Ni/Fe‐MOF nanosheets also showed good electrocatalytic performance toward OER and overall water splitting. The electrolytic cell constructed by Ni/Fe‐MOF achieved a low voltage of 1.55 V (vs RHE) at the current density of 10 mA cm^−2^, which is better than that using Pt/C cathode and IrO_2_ anode (Figure 15f).^278^ Cobalt dithiolene, a molecular catalyst with high HER catalytic activity, has been also integrated into 2D MOF nanosheets to form 2D MOF‐based composites, i.e., Co‐benzenehexathiol (BHT) nanosheets and Co‐2,3,6,7,10,11‐triphenylenehexathiol (THT) nanosheets,^187^ which exhibited good catalytic activity and stability toward HER, under acidic conditions. Similarly, Co‐THT/2,3,6,7,10,11‐triphenylenehexamine (THA) MOF nanosheets, which possess molecular active centers of CoS_2_N_2_, CoS_4_, and CoN_4_, were synthesized for HER.^279^ Besides, LD MOF‐derived materials, such as 1D Co*_x_*Ni*_y_*P nanotubes^237^ and 2D Co/N‐doped carbon nanosheets,^246^ were also active electrocatalysts for H_2_ evolution.

In addition, LD MOF‐based materials have also been severed as electrocatalysts for ORR. For example, Dinca and co‐workers reported a conductive 2D layered MOF, i.e., Ni_3_(2,3, 6,7,10,11‐hexaiminotriphenylene (HITP))_2_, and then used as catalyst for ORR in alkaline electrolyte.^280^ The polarization curve depicts that an onset potential of 0.82 V (vs RHE) was achieved for Ni_3_(HITP)_2_ electrode measured in the O_2_‐saturated solution, which is competitive with most of nonprecious metal catalysts. Besides, LD MOF derivatives, such as P‐doped C nanofibers,^112^ Co‐N/C nanosheets,^71^, ^152^ nanoporous Fe‐N/C composites^249^, ^281^, ^282^ and porous CeO_2_‐Co‐N/C nanosheets,^283^ have been synthesized for high‐efficiency ORR. For instance, Xu and co‐workers used btcpb, a nitrogen‐rich organic molecule, as organic ligand to form 2D Fe‐btcpb nanosheets.^249^ In the subsequent calcination process, a large amount of N atoms in the ligand were converted to Fe‐N active sites for ORR. The catalyst obtained at 700 °C exhibited the highest ORR activity with onset potential of 956 mV in 0.1 m KOH, which is comparable to that of Pt/C catalyst (961 mV).

LD MOF‐based materials were also reported as outstanding electrocatalysts in urea oxidation reaction (UOR) and CO_2_ reduction reaction (CO_2_RR). For example, Qiao and co‐workers prepared Ni‐BDC MOF nanosheet with thickness of ≈4.3 nm via a sonication‐assisted solution method.^284^ High oxidation state of Ni species along with high density of catalytically active sites in the obtained Ni‐BDC MOF nanosheet endow them with excellent UOR performance in 1 m KOH, which only required a potential of 1.36 V (vs RHE) to gain a current density of 10 mA cm^−2^. The overpotential is much lower than that of commercial Pt/C (1.64 V). In another case, cobalt‐metalated porphyrin, an efficient molecular electrocatalyst for CO_2_RR, was used as linker unit to construct Al_2_(OH)_2_TCPP‐Co MOF thin film.^285^ The MOF film with thickness of 30– 70 nm achieved a selectivity of 76% for CO at ‐0.7 V (vs RHE), and its catalytic performance was stable for more than 7 h. In addition, it was demonstrated that the cobalt centers in the MOF film were reduced from Co(II) to Co(I) during the electrochemical process.

#### 4.1.2 Other Catalysis

Heterogeneous catalysis is one of the earliest proposed applications for MOF‐based materials.^286^, ^287^, ^288^, ^289^, ^290^, ^291^ The catalytic active sites of MOF‐based materials are usually originated from their coordinatively unsaturated metal sites (CUSs), and functional organic linkers. Besides, some catalytically active materials which are confined/encapsulated in pores of MOFs, such as polyoxometalates, enzymes, and noble metal NPs, also render those MOFs catalytic active. LD MOF‐based materials with high surface area and abundant accessible active sites on their surfaces have received sufficient attention in the research field of catalysis, including photo‐ and thermo‐driven catalytic reactions.^292^, ^293^, ^294^, ^295^

**Figure 16:**
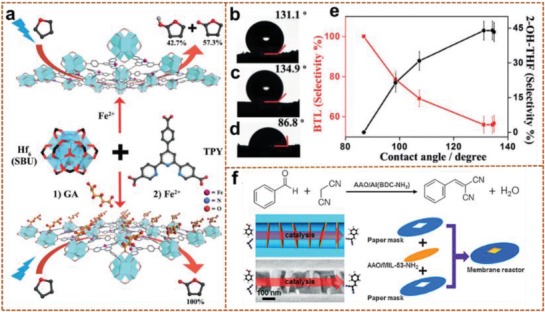
a) Schematic illustration of the modification of the SBUs of 2D Fe^II^/Hf‐TPY nanosheet by using gluconic acid, which regulated the hydrophobicity/hydrophilicity around the active sites of MOFs. Microscopy images of b) water droplets on different Fe^II^/Hf‐TPY films of as‐synthesized film, c) oleic acid‐modified film, and d) gluconic acid‐modified film. e) Relationship between butyrolactone selectivity and contact angles of Fe^II^/Hf‐TPY nanosheet in the aerobic oxidation of tetrahydrofuran. Reproduced with permission.^300^ Copyright 2017, Wiley‐VCH. f) Scheme of the Knoevenagel condensation of benzaldehyde with malononitrile to produce benzylidenemalononitrile, which was catalyzed by the AAO/MIL‐53‐NH_2_ composite membrane. Reproduced with permission.^304^ Copyright 2016, Wiley‐VCH.

Compared with inorganic semiconductor photocatalysts, MOFs have richer selection in light‐harvesting building blocks, making them an ideal platform for photocatalytic reactions.^296^, ^297^, ^298^ For example, Lin and co‐workers reported a series of 2D metal‐organic layer photocatalyst, such as, M(bpy)(CO)_3_X (M = Re and X = Cl or M = Mn and X = Br, bpy = 2,2′‐bipyridine)/Hf_12_‐Ru MOL,^78^ Ir_2_(ppy)_4_Cl_2_/Hf‐4′,6′‐bis(4‐benzoic acid)‐(2,2′‐bipyridine)‐5‐carboxylate (BPY) MOL,^299^ and Ru_2_(bpy)_4_Cl_2_/Zr‐BPY MOL.^177^ These reports clearly indicates improved diffusion of reactive intermediates for MOF nanosheets, as compared to their bulk counterparts. Later, the same group modified 2D Fe^II^/Hf‐TPY MOL by using monocarboxylic acids (e.g., gluconic acid, oleic acid, and caprylic acid) to adjust the hydrophobicity/hydrophilicity around the active sites, thus achieving the regulation of catalytic selectivity for the obtained MOL in the photocatalytic oxidation of tetrahydrofuran (**Figure** **16**a–e).^300^ Recently, a Ni MOF monolayers with strong CO_2_ binding affinity was reported,^294^ CO_2_‐CO conversion was promoted with an effective stabilization of the Ni‐CO_2_ adduct. Peng and co‐workers exfoliated nanosheets from conductive 2D‐MOF Ni_3_(HITP)_2_ and then used them as efficient CO_2_ reduction catalyst under visible light illumination.^295^ Benefiting from the high conductivity of the ultra‐thin nanosheets and more exposed active sites for redox reactions, a high selectivity of 97% for deoxygenative CO_2_ reduction and CO yield rate of 3.45 × 104 µmol·g^−1^ h^−1^ were achieved.

LD MOF‐based materials have also received sufficient attention in the research field of thermo‐driven catalysis. For example, Lee and co‐workers reported a rod‐like Mn(III)‐porphyrin MOF for the oxidation of styrene.^53^ By comparing the catalytic activities of 1D and 3D Cu‐MOF for the C—H activation of diphenylmethane, Wang and co‐workers demonstrated that 1D structure accelerates the catalytic process, due to possessing more accessible active sites.^125^ Moreover, 2D MOF nanosheets have been also widely used in catalysis. For instance, the Lewis acid centers in Co‐BDC nanosheets can catalyze the cycloaddition of CO_2_ with epoxides under normal pressure.^301^ Zr‐porphyrinic MOF nanosheets with thickness of ≈1.48 nm exhibited excellent photocatalytic performance for the oxidation of 1,5‐dihydroxynaphthalene, which is owing to their highly exposed active sites and unique properties of porphyrin units.^79^

In addition to directly acting as catalysts, LD MOFs have been also investigated as support materials for the other catalytically active materials.^59^, ^165^, ^302^ Recently, Lin and co‐workers reported a series of 2D metal‐organic layers, i.e., Fe‐TPY/Zr‐BTB MOL,^64^ CoCl_2_/Hf‐TPY MOL,^178^ FeBr_2_/Hf‐TPY MOL,^178^ which are efficient heterogeneous catalysts for various organic reactions, including hydrosilylation of terminal olefins, C—H amination benzylic, C—H borylation.^64^, ^178^ Moreover, catalytically active NPs can be encapsulated within porous frameworks of LD MOFs to achieve enhanced activity and/or selectivity. For example, Liu and co‐workers reported the encapsulation of noble metal NPs, i.e., Au and Pt NPs, into ZnO@ZIF‐8 rods through a template‐sacrifice method.^116^ By optimization of the growth process for ZIF‐8, the spatial distribution of NPs in MOF can be controlled. It was also found the catalytic efficiency for olefin hydrogenation was enhanced with more Au NPs on the surface of ZnO@ZIF‐8 rods, which is due to minimized diffusion length for reactants. Moreover, benefiting from the molecular sieving behavior of MOFs, this rod‐shaped ZnO@Pt@ZIF‐8 composite exhibited size‐ and shape‐selective to the catalytic substrates. As another example, Wang and co‐workers immobilized Pd clusters on Zn/Ni‐MOF‐2 nanosheet‐assembled hollow nanocubes for the alkoxycarbonylation of aryl halides.^150^ It was revealed that the obtained MOF composite nanosheet can store CO and accelerate the catalytic process. Later on, the same authors prepared Pd NP‐coated hierarchical porous MOF‐5 nanosheets for the reduction of nitroarene.^303^

In order to avoid agglomeration of LD MOF‐based catalyst during catalytic process, the construction of hierarchical structure that assembled by LD MOFs is an effective way, such as 1D@2D hierarchical arrays^226^ and 3D hierarchical nanoflowers.^66^ Recently, Zhang and co‐workers synthesized two different 2D MOF nanoplates, i.e., MIL‐53‐NH_2_ and Al_2_(OH)_2_(TCPP), within the channels of anodized aluminum oxide (AAO).^304^ The immobilization of AAO channels and the 2D structure of MOF nanoplates offer the obtained MIL‐53‐NH_2_/AAO composites excellent catalytic activity and stability in the Knoevenagel condensation reaction (Figure 16f). In view of the poor stability of MOFs in the catalytic process, an effective approach is to transform them to metal oxides and/or carbon‐based composites with better electrochemical stability.^142^, ^250^ For instance, after a high‐temperature pyrolysis process, Ru/Zn‐BTC MOF fibers were converted into Ru/carbon nanofibers, which exhibited high stability in the liquid‐phase hydrogenation of levulinic acid to γ‐valerolactone at 150 °C.^222^ Similarly, 1D CuO/TiO_2_ rod^238^ and 2D Cu_2_O/N‐doped carbon photocatalyst^250^ were obtained by the thermolysis of their corresponding MOF precursors.

### 4.2 Energy Storage

LD MOFs with ultrahigh specific surface area and well‐defined pore structure benefit fast electron and ion transportation, showing enormous potential for electrochemical energy storage devices, such as lithium‐ion batteries (LIBs), metal‐air batteries and supercapacitors. For example, 1D Co‐polycarboxylate (CoCOP) MOF nanowires prepared by a hydrothermal method were explored as electrode of LIBs.^141^ The CoCOP nanowires exhibited a high reversible capacity of 1107 mA h g^−1^ at a current density of 20 mA g^−1^, and a long‐cycling life for more than 1000 cycles at 1 A g^−1^. The lithium storage performance of CoCOP nanowires can be ascribed to the reversible conversion between Co^2+^ and Co^0^ in the MOF. In addition, porous metal oxides obtained by pyrolysis of the specific LD MOFs have attracted intensive attention as electrodes for LIBs in the past years. For instance, porous 1D Co_3_O_4_ rods and 2D GeO_2_ nanosheets were obtained by annealing of rod‐like Co‐MOF‐74 and sheet‐like Ge‐MOF precursors, respectively, and then served as electrode materials for LIBs.^230^, ^305^ In another case, mesoporous ternary metal oxide nanorods, i.e., NiCo_2_O_4_ and Ni_0.3_Co_2.7_O_4_, were prepared from rod‐shaped Co/Ni bimetallic MOF.^72^ Owing to the synergistic effect in the ternary metal oxide, the initial discharge capacity of Ni_0.3_Co_2.7_O_4_ rod reached 1737 mA h g^−1^ at 0.1 A g^−1^, which is much higher than the theoretical capacities of both Co_3_O_4_ (890 mA h g^−1^) and NiO (718 mA h g^−1^). Besides, LD metal oxide and sulfide‐based composites, such as NiFe_2_O_4_/Fe_2_O_3_ nanotubes,^240^ Te@ZnCo_2_O_4_ nanofibers,^113^ MnO/C nanorods,^232^ ZnO@ZnO quantum dots/C nanorod arrays,^114^ Co/CoS_2_/CNT nanotubes,^65^ and MoO_2_/N/C nanopetals,^84^ have also been synthesized by calcination of corresponding LD MOF‐based precursors recently. Owing to the compositional and morphological advantages, these LD metal oxide/sulfide‐based composites presented superior specific capacities and distinguished cycling stabilities, when evaluated as anode electrodes for LIBs.

**Figure 17:**
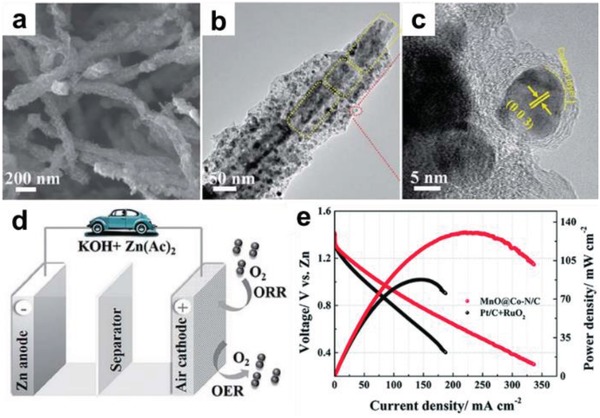
a) SEM, b) TEM, and c) HRTEM images of MnO@Co–N/C composite nanowires. d) Schematic illustration of a fabricated Zn–air battery. e) Discharge polarization curves and corresponding power densities of the Zn–air battery. Reproduced with permission.^243^ Copyright 2018, Royal Society of Chemistry.

Benefiting from the high theoretical energy density, safety and low cost, rechargeable Zn‐air batteries have been considered as promising energy storage device for practical usage. It is well known that the lack of stable and effective OER and ORR bifunctional catalysts for air cathode is one of the main problems encountered by Zn‐air batteries nowadays.^306^, ^307^, ^308^, ^309^ LD MOFs with large surface area and tunable structure provide an avenue for the development of high‐performance Zn‐air batteries.^71^, ^111^ For example, the air cathode based on nanoporous Fe‐N/C nanosheets derived from 2D Fe‐btcpb nanosheets delivered a specific capacity of 727 mA h g^−1^ at 5 mA cm^−2^.^249^ The corresponding energy density reached 965 W h kg^−1^, which is higher than that of Zn‐air battery using Pt/C as cathode. In another example, 1D bamboo‐like MnO@Co–N/C nanowires were prepared by pyrolyzing MnO_2_@ZIF‐67 precursors (**Figure** **17**a–c).^243^ Due to the high electrical conductivity of the 1D nanostructure and the synergistic effect between MnO and porous Co–N/C, the composite nanowires exhibited good performance in rechargeable Zn‐air batteries. Specifically, the MnO@Co–N/C nanowire‐based Zn‐air battery showed a peak power density of 130.3 mW cm^−2^, and a current density of 300 mA cm^−2^ at 0.4 V (Figure 17d,e), exceeding those of Pt/C‐RuO_2_ (1:1)‐based Zn‐air battery (188 mA cm^−2^ and 88 mW cm^−2^). Moreover, MnO@Co–N/C nanowire‐based rechargeable Zn‐air battery shows no obvious voltage change over 633 h (1900 cycles).

LD MOF‐based materials have also been applied as supercapacitor electrodes. For instance, Wei and co‐workers reported the synthesis of layer‐stacked Co‐BDC MOF nanosheets, which exhibited a maximum capacitance of 2564 F g^−1^ at a current density of 1 A g^−1^.^310^ Moreover, the Co‐BDC MOF nanosheets showed good cycling stability with 95.8% retention of the initial capacitance after 3000 cycles at a current density of 2 A g^−1^. The superior supercapacitor performance of Co‐BDC MOF nanosheets may be ascribed to their unique layered structure, and electrically conductive network. Zheng and co‐workers further prepared oriented Co/Ni‐BDC MOF nanosheets on carbon fiber paper (CFP).^311^ It exhibited a specific capacitance of 569 F g^−1^ at a high current density of 32 A g^−1^, which is much higher than that of Co/Ni‐MOF powder. Similarly, the conductive MOF nanowire arrays of Cu‐CAT grown on carbon paper showed a high areal capacitance of about 22 µF cm^−2^, when used as electrodes for solid‐state supercapacitor, which is owing to the high porosity and excellent conductivity of electrode material.^89^ In addition, LD MOFs have also acted as versatile precursors for the preparation of carbon‐based materials and porous metal oxide/sulfide/selenide‐based electrode materials for supercapacitors.^73^, ^140^, ^169^, ^175^, ^239^, ^241^, ^258^, ^259^

LD MOF‐based materials and their derivatives have also been used in other electrochemical energy storage devices, such as sodium‐ion batteries (SIBs), lithium sulfur batteries, and battery‐supercapacitor hybrid devices.^245^, ^248^, ^312^ For instance, Chen and co‐workers reported a layer‐stacked Ni‐BDC MOF nanosheet, which was used as cathode for alkaline battery‐supercapacitor hybrid devices in a mixed electrolyte of 3 m KOH and 0.1 m K_4_Fe(CN)_6_.^312^ The unique layered structure of the Ni‐BDC MOF nanosheet with interlayer distance of about 0.95 nm allows the fast diffusion and intercalation/deintercalation of electrolyte ions, which delivered a high energy density of 55.8 W h kg^−1^ and superior power density of 7000 W kg^−1^. Furthermore, the MOF nanosheet exhibited long‐time cycling stability with a capacitance retention of 90.6% after 3000 consecutive cycles at 10 A g^−1^.

### 4.3 Gas Adsorption and Separation

MOF‐based materials with high surface area, large void spaces and controllable surface properties have attracted much attention in the field of gas adsorption and separation, such as CO_2_ capture, H_2_ and CH_4_ purifications, and CH_4_ storage.^70^, ^313^, ^314^, ^315^, ^316^, ^317^ Previous researches have demonstrated that LD MOFs with high aspect ratio and unique physicochemical properties possess superior sorption behaviors over their bulk counterparts.^68^, ^82^, ^163^ For instance, Marmottini and co‐workers have reported a 1D phosphinate‐based MOF rods for CO_2_ uptake.^127^ At 195 K, the 1D MOF rods exhibited a CO_2_ uptake capacity of 100 mg g^−1^, which is much higher than the capacity of their bulk counterpart. Moreover, the CO_2_ adsorption rate of the 1D MOF rods is five times higher than that of bulk MOF, owing to their unique 1D nanostructure. As another example, Li and co‐workers synthesized a Cd‐TBAPy rod, namely ROD‐8, by the reaction of H_4_TBAPy and Cd(NO_3_)_2_·4H_2_O in a mixed solvent of DMF, dioxane and water.^92^ After removing the guest molecules, the activated ROD‐8a was obtained, which exhibited high uptake capacities of 84 and 143 cm^3^ g^−1^ for CH_4_ and CO_2_, respectively. The ideal adsorbed solution theory (IAST) predictions showed the selective adsorptions of CH_4_ over N_2_, and CO_2_ over N_2_ at 298 K for these activated rod MOF. In addition, 2D MOF nanosheets were also used as excellent gas adsorbents. For example, ultrathin Zr‐BTB nanosheets with thickness of ≈3 nm were prepared via a continuous microdroplet flow reaction.^176^ Owing to the larger external surface, the obtained ultrathin Zr‐BTB nanosheets exhibited high CO_2_ and CH_4_ adsorption performance, which is superior over the thicker Zr‐BTB nanosheets (thickness of ≈49 nm).

**Figure 18:**
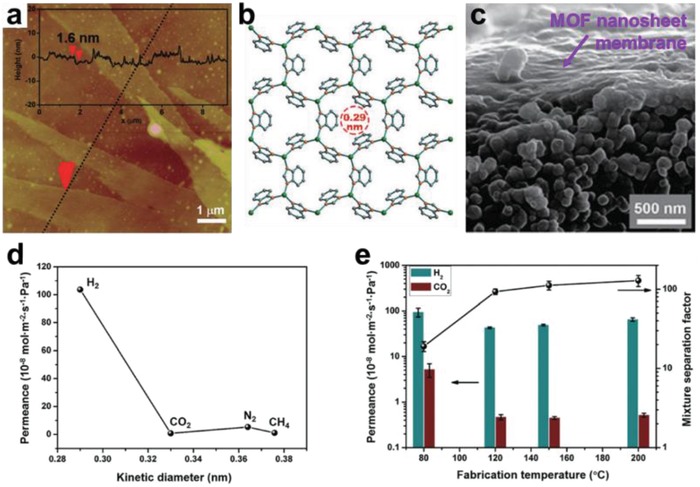
a) AFM image and b) crystal structure of Zn_2_(Bim)_3_ nanosheets, where the green symbol presents Zn, the orange one stands for N, and the gray one is for C. c) Cross‐sectional SEM image and d) single gas permeation of a Zn_2_(bim)_3_ nanosheet membrane obtained at 200 °C. e) Binary gas separation performance of equimolar H_2_/CO_2_ of the obtained Zn_2_(bim)_3_ nanosheet membranes at different temperatures. Reproduced with permission.^67^ Copyright 2017, Wiley‐VCH.

Recently, membrane‐based molecular sieves have attracted increasing attention because of their high effectiveness and low energy consumption. For instance, Gascon and co‐workers reported a bottom‐up synthetic strategy for Cu‐BDC MOF nanosheets, which were then dispersed into a polymer matrix of polyimide (PI) and obtain the MOF‐polymer composite membrane with excellent separation performance for CO_2_/CH_4_ gas mixtures^82^ In another example, 1 nm thick Zn_2_(bim)_4_ nanosheets were prepared via a soft‐physical process, which were then deposited onto α‐Al_2_O_3_ substrate to form the Zn_2_(bim)_4_/α‐Al_2_O_3_ membrane.^160^ The membrane achieved H_2_ permeance of 3760 gas permeation units (GPUs) with a high H_2_/CO_2_ selectivity of 291, and a long‐term stability after testing for 120 h at 150 °C. Later on, the same group reported a sub‐10 nm thick ultrathin Zn_2_(bim)_3_ nanosheet‐based membrane for H_2_/CO_2_ separation.^67^ The Zn_2_(bim)_3_OH nanosheets with thickness of about 1.6 nm were prepared by a top‐down exfoliation method (**Figure** **18**a). The honeycomb‐like aperture with size of about 0.29 nm allows H_2_ (0.289 nm) to pass through, but restricts gases with larger sizes, such as CO_2_ (0.33 nm), from passing through, endows it with good H_2_/CO_2_ separation performance (Figure 18b). After a hot‐drop coating process, MOF nanosheets were firmly covered on the surface of α‐Al_2_O_3_ substrate, and the membrane was obtained (Figure 18c), which achieved a high separation factor (SF) of up to 166 for H_2_/CO_2_ separation. The high performance is owing to the well‐defined size exclusion effect of Zn_2_(Bim)_3_OH nanosheets, which was further confirmed by single gas permeation results (Figure 18d). Interestingly, the authors found that the SF is related to the coating temperature, which affected the interlayer space in the membrane, and leaded to different CO_2_ permeation rate (Figure 18e). Recently, many other LD MOF material‐based membranes with excellent gas adsorption and separation performances were developed, such as Zn_2_(bIm)_4_ nanosheet/GO membrane,^153^ [Cu_2_(ndc)_2_(dabco)]*_n_* nanosheet/polybenzimidazole membrane,^318^ and Cu‐BDC nanosheet/PIM‐1 membrane.^319^

### 4.4 Sensing

Owing to their unique structural characteristics and diversity, MOFs have also exhibited potential applications in sensing various analytes, such as biomolecules, ionic species and environmental toxins.^320^ The sensing performance of MOFs is affected by the diffusion rate of analytes within pores of MOFs.^321^ LD MOFs with high specific surface area and highly exposed active sites are ideal sensing platforms.^75^, ^322^ For example, Zhu and co‐workers reported the fabrication of Cd‐BTC MOF nanotube for trace‐level detection of nitroaromatic explosives.^105^ After the MOF nanotube was placed in 2,4‐dinitrotoluene saturated vapor for 10 s, the fluorescence quenching percentage reached 72.5%. The response rate of Cd‐BTC nanotube for 2,4‐dinitrotoluene vapor was also among the highest values for reported fluorescence‐based chemical sensing materials, which is due to their unique composition and structural advantages of LD MOFs. Similarly, Qian and co‐workers reported a MOF nanosheets, i.e., Ti_2_(HDOBDC)_2_(H_2_‐DOBDC) (NTU‐9‐NS), for highly sensitive and fast‐response luminescent sensing of Fe^3+^, by utilizing their fluorescence quenching effect.^323^ The excellent luminescent sensing ability of NTU‐9‐NS could be ascribed to highly accessible active sites on the surface of nanosheets. Recently, the exfoliated ZSB‐1 nanosheets were also used as fluorescence sensor to detect Fe^3+^ ions.^80^ The ultrathin ZSB‐1 nanosheets showed a low detection limit for Fe^3+^ ions (0.054 × 10^−6^ m), which is much lower than that of the bulk counterpart (0.110 × 10^−6^ m). In addition to fluorescence quenching, the fluorescence turn‐on response of LD MOF materials was also investigated for detection of specific analytes, such as uric acid and volatile organic compounds.^69^, ^324^ As a typical example, a bio‐friendly Pb(II)‐based MOF nanotube was developed for uric acid detection.^69^ The host‐guest interactions of the MOF nanotube with uric acid result in responsive turn‐on fluorescence, delivering high selectivity for uric acid with a low detection limit of 4.3 × 10^−3^ m.

**Figure 19:**
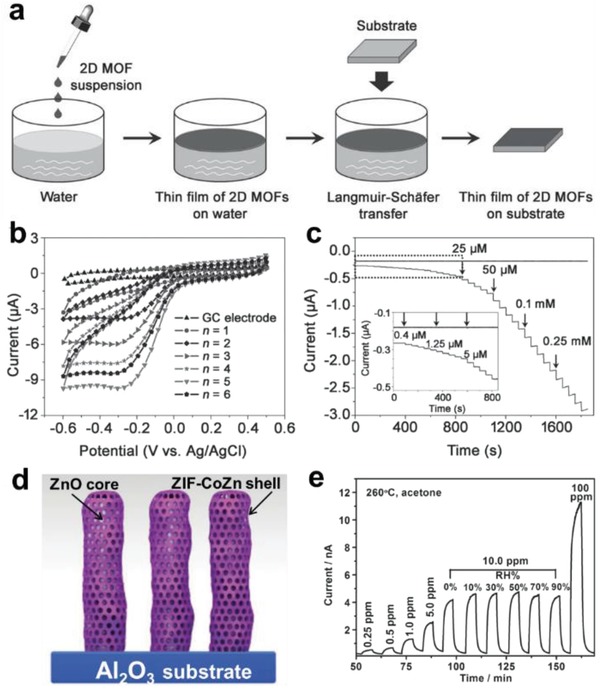
a) Schematic illustration of the preparation process for the 2D MOF nanosheet‐based thin films. b) Cyclic voltammetry curves at a scan rate of 50 mV s^−1^ and c) typical amperometric response at an applied potential of −50 mV of bare GC electrode and various GC/(Co‐TCPP(Fe))*n* (*n* = 1–6) electrodes in 0.1 m PBS (pH 7.4) with 0.5 × 10^−3^ m H_2_O_2_. Reproduced with permission.^168^ Copyright 2017, Wiley‐VCH. d) Schematic illustration of the ZnO@ZIF‐CoZn core‐sheath nanowire array. e) Gas sensing properties of ZnO@ZIF‐CoZn toward acetone vapor. Reproduced with permission. Reproduced with permission.^115^ Copyright 2016, Wiley‐VCH.

Besides, LD MOFs can be also used as electrochemical sensors because of their high surface area and outstanding adsorption abilities. For example, Zhang and co‐workers first prepared ultrathin 2D MOF nanosheets, i.e., Co‐TCPP(Fe), through a surfactant‐assisted method.^168^ Then, the nanosheets were transferred to substrates, such as Si wafer or glassy carbon (GC), to form 2D MOF‐based thin films, which were used as electrochemical platforms to detect H_2_O_2_ (**Figure** **19**a). The GC/Co‐TCPP(Fe) electrode exhibited heme protein–like activity with a detection limit of 0.15 × 10^−6^ m toward H_2_O_2_, which is better than those of natural heme protein‐based sensors (Figure 19b–c). Moreover, a series of conductive 2D MOFs, including Cu_3_(HITP)_2_, Cu_3_(HHTP)_2_ and Ni_3_(HITP)_2_, were also prepared and used for detection of ammonia and volatile organic compounds (VOCs).^86^, ^325^

It has been also demonstrated that the smart combination of other functional materials with LD MOFs can achieve improved sensing performance. For example, a hydrophobic MOF layer of ZIF‐CoZn was coated on the surface of ZnO nanowire to form a core‐shell nanowire array, and then used for chemiresistor sensor (Figure 19d).^115^ It was found that the selectivity between acetone and humidity of the sensor has been significantly improved after coating with a layer of ZIF‐CoZn. The response current of ZnO@ZIF‐CoZn increased with the concentration of acetone, but remained unchanged with increase of relative humidity (Figure 19e). Recently, LD MOF‐based composites, such as ZnO@ZIF‐8 nanorod, Ti@TiO_2_/CdS/ZIF‐67, and Au nanocluster/521‐MOF nanosheet, have been also developed and used for detecting H_2_O_2_ 74, 277 and cocaine.^326^

In addition, LD MOF‐derived porous carbon and metal oxides with high specific surface area have been also used to construct high‐performance sensors. For instance, the Pd@ZnO‐WO_3_ nanofibers, which were prepared by annealing the Pd@ZIF‐8/PVP/ammonium metatungstate hydrate composite fibers, exhibited high sensitivity (*R*_air_/*R*_gas_ = 4.37 to 100 ppb) and fast response speed (≈20 s) for toluene detection.^327^ As another case, Yamauchi and co‐workers reported the fabrication of nanoporous carbon fibers by using Al‐based MOF as precursor through a calcination‐etching process, which exhibited good sensing capabilities toward toxic aromatic guests.^234^

### 4.5 Other Applications

In addition to the aforementioned applications, LD MOF‐based materials have also been applied in other fields, such as drug delivery,^52^, ^104^ nanofiltration,^328^, ^329^ and optical applications.^83^ For instance, the 1D worm‐like ZIF‐8 nanotubes showed high loading capacity and pH‐sensitive release property for the anticancer drug doxorubicin.^104^ The MOF films constructed by Cu‐tri(β‐diketone) nanosheets were demonstrated to have excellent size‐selective separation properties for Au NPs, which allowed the filtration of Au NPs with a cut‐off size of ≈2.4 nm.^328^ Recently, temperature‐dependent charge transport properties^88^ and morphology‐dependent luminescence properties^330^ of LD MOFs have been revealed, which may open up a new avenue for the applications of LD MOF‐based materials.

## 5 Conclusions

In this review, the recent research progress of LD MOF‐based materials and their derivatives has been summarized. Compared to bulk MOF crystals, LD MOFs have advantages of unique flexibility and high density of surface‐active sites, as well as the distinct physicochemical properties. The unique properties of LD MOFs have attracted wide attention and shown promising performance in many fields such as catalysis, energy storage, and sensing. For example, as heterogeneous catalysts, LD MOFs can be catalyzed by their own metal nodes or functionalized organic ligands. Besides, owing to their porous nature, LD MOFs are capable of acting as carriers for loading of catalytically active nanoparticles. Moreover, like other LD nanomaterials, 1D or 2D MOFs can be used as building blocks to construct hierarchical structured materials, such as a macro assembly composed of MOF nanosheets, which generates new function or improved properties.

Up to now, a variety of strategies have been developed, including the usage of rationally designed SBUs and organic ligands, restriction of the growth of specific crystal faces, thermodynamic and kinetic regulations of the growth process, and the top‐down exfoliation method, etc. Through the various state‐of‐the‐art methods, numerous LD MOFs, such as 1D MOF nanowires, nanotubes, nanorods and 2D MOF nanosheets, have been successfully prepared and explored for a wide range of applications.

In recent years, MOFs have been also proven to be versatile precursors for the convenient preparation of various derivatives, such as metal oxides/sulfides/phosphates and porous carbon‐based composites. Using LD MOF‐based materials as the precursors, a number of derivatives with 1D, 2D and more complex nanostructures can be readily obtained. These derivatives not only retain the porosity brought from MOF precursors, but also possess unique properties from LD structures, showing great promising in the various fields, such as catalysis and energy storage.

Despite great efforts have been made in the preparation and applications of LD MOF‐based materials, this research field is still in its infancy. The main challenges of this research area are discussed as follows. 1) High‐quality and scalable preparation of LD MOF‐based materials remains a great challenge, especially for the preparation of LD MOF/MOF composites. There are few reports on precise control of the growth orientation of MOF crystal on another LD MOF matrix. 2) Poor structural stability in humid air or aqueous solutions is a well‐known issue for the practical applications of MOFs.^331^ LD MOFs usually possess more exposed surfaces compared to their bulk counterparties, which makes them more sensitive to humidity and leads to a further reduction in stability. Although improved stability of MOF‐5 and HKUST‐1 was achieved by coating it with a hydrophobic layer of poly(dimethylsiloxane) (PDMS),^332^ such approach is not a general solution to the majority of MOFs. Therefore, developing novel protection way to enhance stability of LD MOFs is of great significance for their practical applications. 3) The high tendency to form agglomeration is another issue for LD MOFs, which is resulted from their high surface energies, large aspect ratios, and small sizes. Although it is an effective way to avoid agglomerations of LD MOFs by growing them on substrates (e.g., nickel foam and carbon paper) and construct 1D nanowire arrays or 2D nanosheet arrays, such preparation strategy generally has poor universality. 4) Converting LD MOF‐based materials into porous derivatives endows them with various new functions. However, the unique structural characteristics of LD MOFs lead to inevitable agglomerate during heat treatment, resulting in the loss of active sites. A general and efficient methods to convert MOFs into high‐performance LD derivatives is still required.

## Conflict of Interest

The authors declare no conflict of interest.
